# Supplementary material for: Antibody response to tetanus, diphtheria, poliomyelitis, hepatitis B, and H. influenzae b vaccines in allogeneic hematopoietic stem cell transplant adult recipients: A multicenter trial
Source: PLoS One. 2025 Oct 27;20(10):e0335224. doi: 10.1371/journal.pone.0335224 (PMC12558451; doi:10.1371/journal.pone.0335224)
Supplement: Attachment — Submitted filename: Hexavalent vaccines for immunization.pdf [file pone.0335224.s001.pdf]

REVIEW 10.1111/1469-0691.12444

Hexavalent vaccines for immunization in paediatric age

S. Esposito, C. Tagliabue, S. Bosis, V. Ierardi, M. Gambino and N. Principi

Paediatric High Intensity Care Unit, Department of Pathophysiology and Transplantation, Università degli Studi di Milano, Fondazione IRCCS Ca' Granda Ospedale Maggiore Policlinico, Milan, Italy

Abstract

Despite the potential for protection against a broad spectrum of pathogens, the availability of an increased number of effective vaccines could lead to a significant reduction in vaccination coverage as the result of issues with implementation of new vaccines within existing protocols. To overcome these problems, the development of combined vaccines has been promoted. The use of combined vaccines offers a number of potential benefits, including a reduction in the number of patient visits, reduced complications associated with multiple intramuscular injections, decreased costs of stocking and administering separate vaccines, and a lowering of the risk of delayed or missed vaccinations. The hexavalent vaccine includes antigens against diphtheria, tetanus, acellular pertussis (DTaP), hepatitis B (HBsAg), poliomyelitis (P1, P2, P3) and *Haemophilus influenzae* type B (Hib) infections. The primary goal of this review is to discuss the immunogenicity, efficacy, safety and tolerability of several hexavalent preparations that are either commercially available or still under development.

Keywords: Anti-HBs, combined vaccines, hepatitis B, hexavalent vaccine, pertactin, pertussis
Article published online: 19 December 2013
Clin Microbiol Infect 2014; **20** (Suppl. 5): 76–85

Corresponding author: S. Esposito, Paediatric High Intensity Care Unit, Department of Pathophysiology and Transplantation, Università degli Studi di Milano, Fondazione IRCCS Ca' Granda Ospedale Maggiore Policlinico, Via Commenda 9, 20122 Milano, Italy
E-mail: susanna.esposito@unimi.it

Introduction

Over the last 20 years a significant number of new effective vaccines against infectious diseases have become available. Most of these have been adopted worldwide for use in children, with a relevant increase in the complexity of paediatric immunization schedules [1]. However, these regimens also increase the number of injections administered in a single visit, leading many immunization providers and parents to refuse one or more immunizations because of the child's fear of needles [2] and pain [3–7] in addition to a number of unsubstantiated concerns regarding safety [8,9]. Consequently, the availability of more vaccines could lead to a significant reduction in vaccination coverage through greater difficulties in implementing programmes for new vaccines. To overcome these issues, the development of combined vaccines was promoted. The use of combined vaccines, which include several antigens in a single administration, have a number of potential benefits including a reduction in the number of visits and complications related to multiple intramuscular injections, decreased costs of stocking and administering separate vaccines, and reduced risk of delayed or missed vaccinations [10]. The combined diphtheria, tetanus and pertussis vaccine, which includes a whole cell pertussis component (DTwP) or two or more pertussis antigens (DTaP), has already been incorporated into the national immunization schedules of several countries worldwide. This served as the core formulation to which other vaccines were added. To gain acceptance by health authorities, combination products had to demonstrate that their use was not associated with any significant decrease in immunogenicity or efficacy, or increase in reactogenicity with any component compared with the individual vaccine given separately [11].

One formulation known as the hexavalent vaccine combines DTaP with antigens against hepatitis B (HBsAg), poliomyelitis (P1, P2, P3) and *Haemophilus influenzae* type B (Hib) infection. A number of similar but not identical hexavalent preparations have been developed by pharmaceutical companies. The primary goal of this review is to discuss the immunogenicity, efficacy, safety and tolerability of several hexavalent preparations that are either commercially available or still under development.

©2013 The Authors
Clinical Microbiology and Infection ©2013 European Society of Clinical Microbiology and Infectious Diseases

Hexavac

Hexavac® (Sanofi Pasteur MSD, Lyon, France) was licensed in Europe in October 2000 as a paediatric primary and booster immunization and is widely used in many European countries. One single dose is composed of D toxoid (≥20 IU), T toxoid (≥40 IU), pertussis toxoid (PT) (25 µg), pertussis filamentous haemagglutinin (FHA) (25 µg), HBsAg (produced from recombinant strain of the yeast *Saccharromyces cerevisiae*) (5.0 µg), P1 (Mahoney strain) (40 DAU), P2 (MEF 1 strain) (8 DAU), P3 (Saukett strain) (32 DAU) and Hib (polyribosylribitol phosphate) 12 µg conjugated to tetanus toxoid (24 µg). Several comparative, controlled clinical trials deemed Hexavac to be very effective in assuring long-term protection against all of the indicated target diseases with a high degree of safety and tolerance. It was also deemed non-inferior or equivalent to comparator vaccines, including both separate vaccine components and Infanrix hexa, the second hexavalent vaccine available in this time period [12]. However, in September 2005 the European Medicines Agency recommended suspension of Hexavac marketing authorization because of the reduced immunization properties of the hepatitis B virus (HBV) component [13]. In particular, it was found that although >95% of children vaccinated with Hexavac seroconverted and had protective antibody concentrations (≥10 IU/L) 1 month after primary immunization, 5–20% of them had relatively low antibody titres (≤100 IU/L) [14–16]. Moreover, these children had a significantly diminished response to a subsequent booster dose compared with children with greater antibody titres after the primary series [14–16]. Data confirming the low immunogenicity of the hepatitis B component of Hexavac were further collected when this vaccine was administered in conjunction with the heptavalent pneumococcal conjugate vaccine (PCV7) or with the meningococcal type C conjugate vaccine. In both cases, vaccinated children had lower than expected anti-HB seroconversion rates and antibody geometric mean titres (GMTs) [16,17]. Because peak antibody levels achieved after primary and booster immunizations condition the length of the period during which concentrations remain within the protective range, it was assumed that in a proportion of children immunized with Hexavac the vaccine might not have assured protection against hepatitis B during adolescence and adulthood. Consequently, the vaccine lost authorization for use in children and was withdrawn from the market. Moreover, although the European Medicines Agency did not mandate the immediate revaccination of children that had received Hexavac, some health authorities recommended the administration of a booster dose of hepatitis B vaccine to ensure long-term adequate protection [18].

However, further studies have clarified the characteristics of the immune response evoked by the hepatitis B component of Hexavac, apparently reducing the importance of its lower antibody production for children in the first years of life. Initial surveillance studies did not report evidence of breakthrough HBV infections in children vaccinated with Hexavac. In Italy, a country where this vaccine was largely used, no case was reported between 2000 and 2009, i.e. at least 3–4 years after the last vaccine dose [19]. Moreover, it was demonstrated that in healthy vaccinated children the immunological memory for HBsAg might persist regardless of the presence of protective antibodies, providing effective protection even in those showing waning or undetectable concentrations of anti-HBs after primary vaccination [19–21]. In the study carried out by Zanetti *et al.*, which included 831 children 5–6 years old who had received Hexavac at 3, 5 and 11–12 months of age. The study noted that despite the fact that over 60% of them did not have protective anti-HBs concentrations at the moment of administration of the booster dose, a protective antibody response (≥10 IU/L) was evoked in 92.1% of study participants [21]. This was considered the best evidence that even in the absence of protective antibody levels, children who had received Hexavac maintained T-cell memory and were able to trigger anti-HBs production by B cells when exposed to the viral antigen. The study concluded that because hepatitis B has a long incubation period, the effective immune memory of primed children assures the possibility of developing adequate protection against acute disease and the development of a chronic carrier state, independent of the antibody level [21]. Consequently, a booster dose of hepatitis B vaccine was considered not mandatory in immunocompetent participants who were given Hexavac [21]. However, a recent meta-analysis of studies that have evaluated the persistence of protection after hepatitis B vaccination identified maternal carrier status (OR 2.37; 95% CI 1.11–5.08), administration of a lower vaccine dosage than presently recommended (OR 0.14; 95% CI 0.06–0.30) and the gap time between the last and preceding doses of the primary vaccine series (OR 0.44; 95% CI 0.22–0.86) as determinants for persistence of anti-HBsAg antibodies ≥10 IU/L [22]. A lower vaccine dosage was also associated with failure to respond to booster (OR 0.20; 95% CI 0.10–0.38) [22]. Because Hexavac HBsAg content was only 5 µg, the possibility that children who received this vaccine could be at higher risk of losing immune memory and developing infections of HBV in adolescence or in adulthood cannot be excluded. On the other hand, recent studies showed that immune memory may diminish during the second decade post-vaccination (particularly in children vaccinated at birth), suggesting the need for a booster dose

©2013 The Authors

14690691, 2014, s5, Downloaded from https://onlinelibrary.wiley.com/doi/10.1111/1469-0691.12444 by Jss Medical College, Wiley Online Library on [25/07/2025]. See the Terms and Conditions (https://onlinelibrary.wiley.com/terms-and-conditions) on Wiley Online Library for rules of use; OA articles are governed by the applicable Creative Commons License

during adolescence [23–25]. Wu *et al* [26] showed that 15% of adolescents born to HBsAg/HBeAg-positive mothers who received primary infantile vaccination developed chronic HBV infection. In addition, one in six vaccine recipients were unable to respond to booster vaccination, having lost immunological memory [26].

Further doubts about the use of Hexavac were raised when a possible association between immunization with this vaccine and the occurrence of sudden unexpected death (SUD) was suspected [27]. Three SUDs occurred in Germany between November 2000 and June 2003 in toddlers within 48 h after the administration of the booster dose of the vaccine. No signal was detected for doses given during the first year of life. Standardized mortality ratios for SUD cases within 1 day of vaccination were 31.3 (95% CI 3.8–113.1; two cases observed; 0.06 cases expected) and 23.5 within 2 days after vaccination (95% CI 4.8–68,6; three cases observed; 0.13 cases expected). Despite the fact that these data did not prove a causal relationship between vaccination and SUD, they were considered the basis for the need for intensifying surveillance. In contrast, an Italian case series study including *c.*95% of 3.2 million neonates born in the period 1999–2004 reported that the association between hexavalent vaccine administration and risk of SUD in the first 14 days after vaccine administration was significantly lower than that estimated in Germany (relative risk 1.5) and was limited to the first vaccine dose, at an age when the incidence of SUD is also the highest. Relative risk in the first 2 days after vaccination was 0.7 and 2.3 for Hexavac and Infanrix hexa, respectively, whereas it was 2.8 versus 1.4 and 1.6 versus 1.5 for the first week and for the 2 weeks after vaccine administration [28]. Starting from these data, it was concluded that the limited increase in relative risk appeared to be confined to the first dose and may be partially explained by the confounding effect of age.

In conclusion, although the problems of poor immunogenicity of hepatitis B vaccines and the need for a booster dose during adolescence are still debated, Hexavac was the hexavalent vaccine with which the greatest problems developed; this seems sufficient to justify the decision of the European Medicines Agency. This explains why the vaccine was abandoned and its producers shifted towards the development of a new hexavalent preparation.

Infanrix Hexa

Licensed as Hexavac in 2000, Infanrix® (GSK, Riixensart, Belgium) hexa is the only hexavalent vaccine authorized for paediatric use in Europe. Its composition is similar to that of Hexavac with two main exceptions. In addition to the pertussis antigens PT and FHA, pertactin (PRN 8 μg) is also included. HBsAg is present in a doubled amount, 10 μg instead of 5 μg. Additionally, unlike Hexavac, Hib antigen needs to be reconstituted before use.

Several studies have evaluated the immunogenicity, safety and tolerability of this vaccine after primary immunization (two or three doses in the first 6 months of age according to the schedules recommended for infants) and after a booster dose at 12–15 months of age in comparison with several DTaP-based pentavalent vaccines administered in conjunction with monovalent HBV or Hib vaccines. Several studies have also compared Infanrix hexa with Hexavac [29]. Evaluation of immunogenicity was performed on blood samples drawn 1 month after the last primary series dose and 1 month after the booster administration. Antibody concentrations against D and T toxoids of ≥0.1 IU/mL, HBsAg of ≥10 IU/L, polyribosylribitol phosphate polysaccharide (PRP) of ≥0.15 mg/mL and/or ≥1 mg/mL (markers of short-term and long-term protective immunity, respectively) and against P1, P2 and P3 antigens of ≥1 : 8 were considered the cut-off values to evaluate seroconversion and seroprotection rates [30]. Moreover, because no generally accepted seroprotective antibody levels for pertussis antigens were established, vaccine seroresponse/seropositivity rates against PT, FHA and PRN were assessed and vaccine response was defined as the proportion of patients with post-vaccination antibody titres of ≥5 U/mL in initially seronegative infants and minimal maintenance of pre-vaccination antibody titres in infants who were seropositive before vaccination (i.e. titres ≥5 U/mL). Seropositivity rates were defined as the proportion of infants with antibody titres ≥5 U/mL.

Throughout all of the studies evaluating an immune response it was demonstrated that all components of Infanrix hexa were highly immunogenic and equivalent or non-inferior to comparators (Fig. 1) [31–37]. In particular, seroprotective antibody concentrations against D, T, P1, P2 and P3 were achieved in ≥95% of children after primary series and in ≥99% after booster dose compared with ≥94% and 100% of the children who received comparators [31–37]. Seroprotection was still found in most of the children before the booster dose and this evoked a strong immune response, independently from the schedule and the type of vaccine used for primary immunization. Similar data were reported for the three P antigens. Response rates against PT, FHA and PRN were ≥95% and ≥86%, after primary vaccination and booster dose, respectively, compared with ≥90% and 100% of the children who were given comparator vaccines [30–36]. Short-term protection against Hib was observed in ≥93% and in ≥98% of children who were treated with Infanrix hexa after primary series and booster dose, respectively, in comparison to ≥92% and 100% of infants who received comparator vaccines. Antibody concentrations

©2013 The Authors

14690691, 2014, s5, Downloaded from https://onlinelibrary.wiley.com/doi/10.1111/1469-0691.12444 by Jss Medical College, Wiley Online Library on [25/07/2025]. See the Terms and Conditions (https://onlinelibrary.wiley.com/terms-and-conditions) on Wiley Online Library for rules of use; OA articles are governed by the applicable Creative Commons License

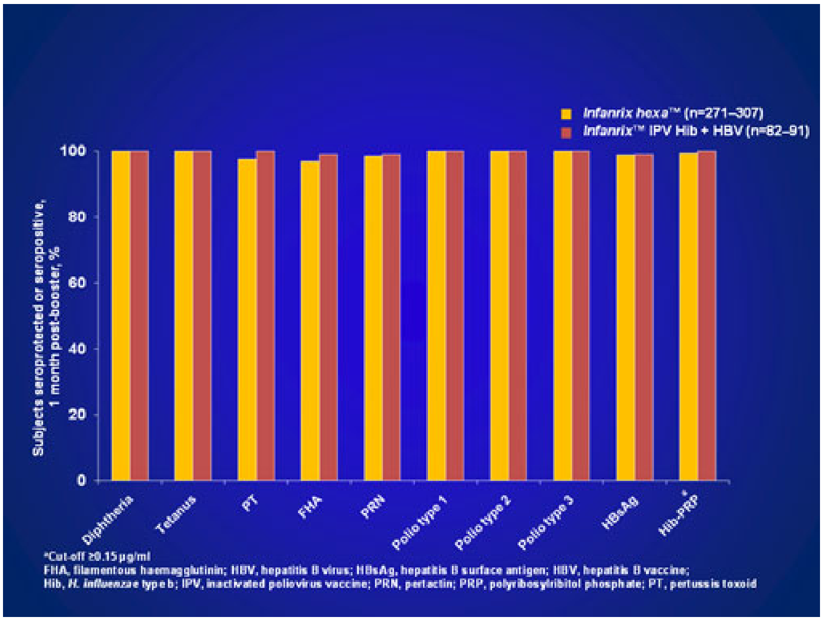

FIG. 1. Immune response evoked by Infanrix hexa in children 15–18 months of age after a booster dose in comparison to Infanrix IPV Hib + HBV. Adapted from Heininger *et al.* [37].

suggestive of long-term protection against this pathogen were achieved in ≥62% and ≥98% of children included in the Infanrix hexa group after primary and booster vaccination, respectively, whereas children who received comparators were protected in ≥66% and ≥97% of the cases. Finally, a strong immune response against HBsAg was observed. Seroprotective antibody concentrations were found in ≥96% of infants after primary series and in ≥98% after booster dose compared with ≥82% and 100% of children who received comparators, irrespective of the vaccination schedule [31–37]. A strong immune response against Infanrix hexa antigens after primary vaccination and booster dose was also evoked in children who were given a dose of hepatitis B vaccine at birth [38,39] as well as in preterm infants [40–42]. Finally, co-administration with other vaccines did not modify the immunogenicity of Infanrix hexa. This was demonstrated with pneumococcal conjugate vaccines, independently from the number of pneumococcal serotypes included in these preparations [43–47], with meningococcal conjugate vaccines, both with preparations including serogroup C [48,49] and with that against serogroups A, C, W135 and Y [50], with measles, mumps, rubella and varicella vaccine [51] and with rotavirus vaccine [52].

Protection evoked by Infanrix hexa was found to persist in the long term (Table 1). In the study carried out by Zinke *et al.*

TABLE 1. Seroprotection/seropositivity rates in children who have received Infanrix hexa as primary series and as a booster dose. Adapted from Zinke *et al.* [53].

Antigen	Subjects seroprotected/seropositive, %		
	Children 4–6 years of age	Children 7–9 years of age (not boosted)	Children 7–9 years of age (boosted)
Diphtheria	96	86	>98[a]
Tetanus	74	64	100[a]
PT	25	32	>60[a]
FHA	>95[a]	98	100[a]
PRN	>85[a]	87	100[a]
Polio type 1	>95	91	100[a]
Polio type 2	>95	91	100[a]
Polio type 3	>95	97	100[a]
HBsAg	86	77	Not tested
Hib-PRP	>95	99	Not tested

FHA, filamentous haemagglutinin; HBsAg, hepatitis B surface antigen; Hib, *H. influenzae* type b; PRN, pertactin; PRP, polyribosylribitol phophate; PT, pertussis toxoid.
[a]Exact numbers not quoted.

©2013 The Authors

14690691, 2014, s5, Downloaded from https://onlinelibrary.wiley.com/doi/10.1111/1469-0691.12444 by Jss Medical College, Wiley Online Library on [25/07/2025]. See the Terms and Conditions (https://onlinelibrary.wiley.com/terms-and-conditions) on Wiley Online Library for rules of use; OA articles are governed by the applicable Creative Commons License

[53] it was demonstrated that children who were administered this vaccine as a primary series with a booster dose in the second year of life had seroprotective/seropositive antibody levels against all vaccine antigens up to *c.*6 years later with the exception of those against PT. In particular, in these children anti-HBsAg antibody concentrations were ≥10 IU/L in 77% and ≥100 IU/L in 33.9% of the children. Moreover, in the group of children 7–9 years old who received a second booster dose at 5–6 years, antibody concentrations against T, FHA, PRN and P1, P2 and P3 and those against D were found to be seroprotective or seropositive in 100% and 98% of the cases, respectively, showing long-term persistence of immune memory against these antigens. Unfortunately, in these children no evaluation of anti-HBsAg antibody concentrations was performed. An anamnestic response was observed in >90% of cases in children aged 4–6 years and in those 7–9 years old who had previously received Infanrix hexa for primary and booster immunization when they were tested with a hepatitis B vaccine [54,55]. Interestingly, immune memory was also induced by previous vaccinations in children that were not considered responders because their anti-HBsAg antibody titres were <3.3 IU/L [55]. More than 82% of these children had antibody concentrations ≥10 IU/L after challenge, despite the fact that the magnitude of post-dose antibody response was directly proportional to the antibody level before the challenge dose and the response to the booster dose achieved in the second year of life [55]. Because vaccination during infancy with adequate HBsAg amount and number of doses was found to assure protective immunity until about 20 years of age, it seems clear that Infanrix hexa could assure long-term protection without any further booster administration during adolescence in immunocompetent participants. However, several recent reports show that in some cases immune memory could wane, indicating that some participants may develop an increased risk of HBV infection despite adequate vaccination during infancy [24,26]. A booster dose should be considered for individuals who have lost immunological memory and live in highly endemic regions where HBsAg carriers are often positive for HBeAg [25].

Epidemiological data clearly indicate that Infanrix hexa is protective against Hib infection. A surveillance study showed that none of the children fully immunized with this vaccine developed invasive Hib during a 5-year observational period [56]. Moreover, it is believed that this preparation could be highly protective against pertussis because it includes the same amount of PT, FHA and PRN contained in Infanrix, a very effective DTaP vaccine. In two studies it was demonstrated that this vaccine was effective in ≥83.9% of the cases against pertussis [57,58]. A 7-year follow-up of children fully vaccinated with Infanrix showed the incidence of pertussis reduced to 8/100 000 person-years relative to a pre-vaccination incidence of 225/100 000 person-years [59].

All of the studies demonstrate that Infanrix hexa is generally well tolerated and safe in children, even in those born prematurely and when it is co-administered with other routine vaccines [29]. Serious adverse events were reported in <3% of vaccine recipients and in most cases they were considered to be unrelated to vaccine administration [33–35,60]. However, transient and mild local and systemic adverse events were common after vaccine administration, although the frequency of these events was not significantly different from comparator vaccines. In the safety and reactogenicity report by Zepp *et al.* [60], redness at the injection site was the most common adverse event. Fever (≥38.0°C) occurred in about one-third of children but the risk of high fever (≥39.5%) was marginal (about 3.5%). Finally, Infanrix hexa was not associated with SUD in the German study by von Kries *et al.* [27] and in a second study [56] carried out in the same country in which the proportion of SUDs observed in a given month and the number of children who were given the vaccine in the same month was evaluated. This last analysis showed that among children receiving the vaccine in the first year of life, the observed number of SUDs within 14 days of vaccination was less than the expected number of cases [56]. Similar findings were observed in children vaccinated in the second year of life [56].

In conclusion, Infanrix hexa has been demonstrated to be immunogenic, effective, safe and well tolerated in children regardless of gestational age at birth, and not significantly different from the vaccines used as comparators. Moreover, its co-administration with other vaccines does not modify its immunogenicity or reactogenicity. Its protection can be considered similar to that offered by Hexavac for all the diseases against which it was prepared, with the exception of hepatitis B. Its immunogenicity seems superior to Hexavac for hepatitis B, as demonstrated in clinical trials by the greater seroprotection rates and higher antibody GMT shown after the primary series, the greater booster response and the persistence of protection until 7–9 years of age in a greater number of children. Moreover, the high immune response evoked in children with poor antibody production after the primary series and booster dose after hepatitis B challenge suggests that protection against this infection can be assured for a significantly longer period of time in the greatest proportion of vaccinated immunocompetent participants. Only poor responders or non-responders may merit a booster dose, particularly when a participant is continuously exposed to HBV. In addition, the presence of PRN in Infanrix hexa offers a potential additional benefit against pertussis in comparison with Hexavac.

©2013 The Authors

14690691, 2014, s5, Downloaded from https://onlinelibrary.wiley.com/doi/10.1111/1469-0691.12444 by Jss Medical College, Wiley Online Library on [25/07/2025]. See the Terms and Conditions (https://onlinelibrary.wiley.com/terms-and-conditions) on Wiley Online Library for rules of use; OA articles are governed by the applicable Creative Commons License

Hexyon

The same pharmaceutical company that produced Hexavac has developed a new hexavalent vaccine, Hexyon® (Sanofi Pasteur MSD, Lyon, France). It has the same composition against D, T, P, polio and Hib, but differs in hepatitis B content. Instead of 5 µg HBsAg produced from recombinant strain 2150-2-3 of the yeast *Saccharromyces cerevisiae*, it includes 10 µg of HBsAg produced in the yeast *Hansenula polymorpha*. Hexyon has been evaluated in several clinical trials and is currently registered in markets outside Europe for the primary immunization of children from 6 weeks of age and for booster vaccination up to 24 months of age [61,62]. It is a liquid vaccine that does not require reconstitution of any component before injection, facilitating administration and reducing the risk of medication error. The immunogenicity of Hexyon used for primary vaccination has been evaluated after three doses of the vaccine, according to the recommended immunization schedules of the countries in which the trials were conducted. In particular, the vaccine was given at 6, 10 and 14 weeks of age in South Africa [63], at 2, 3 and 4 months in Turkey [64] and at 2, 4 and 6 months of age in Argentina [65], Columbia, Costa Rica [66], Mexico, Peru [67,68] and Thailand [69]. When Hexyon was evaluated before and after booster dose, it was administered at 15–18 months of age [64,67,70]. In all primary series trials [64–70], Hexyon immunogenicity and safety were compared with standard doses of licensed vaccines. Quadrivalent, pentavalent and hexavalent vaccines were used as comparators, with the addition of monovalent vaccines lacking in the combined preparations. Antibody concentrations against D, T, PT, FHA, P1, P2, P3 and Hib were generally evaluated with the same criteria and methods previously described for Infanrix hexa with the exception of pertussis antigens, for which an four-fold or greater increase from baseline in antibody concentration was predefined as the surrogate measure of seroconversion. Immune response was systematically similar to that obtained with comparators. Among the children who had received Hexyon as a primary immunization, the vaccine evoked seroprotective levels of antibody against D and T in ≥95% and 100% of the cases, respectively [64–70]. Moreover, seroprotective levels against P1, P2, P3 and Hib were reached in ≥98%, ≥95%, ≥97 and ≥91% of cases, respectively [64–70]. Although there is a limit to the absence of PRN, seroconversion rates for acellular pertussis antigens were ≥90% for PT and ≥82% for FHA [64–70]. After the booster dose, seroprotection rates with Hexyon were consistently 100% for P1, P2, P3 and Hib [64–70]. For these antigens, GMTs of antibodies were generally higher than those following primary immunization. Similar results were obtained for D and T, whereas seroconversion rates for PT and FHA were ≥92% and ≥87%, respectively [64–70]. However, while GMTs for D and T after the booster were generally higher than after the primary series, those for PT and FHA were similar or only slightly higher than those obtained after the priming series [64–70].

It was found that seroconversion and seroprotection rates in response to HBSg antigen after primary series of Hexyon were always high and not inferior to those induced by comparators, independent of the schedule of administration. However, as expected, children who have received a dose of hepatitis B vaccine at birth had antibody GMT greater than those without this prophylaxis, although the percentage of those seroprotected was similar (>94%). Table 2 shows the four studies in which Hexyon was compared with Infanrix hexa for primary immunization. Two of these studies were carried out in children who were immunized at 2, 4 and 6 months of age without any hepatitis B vaccine at birth and without other simultaneous vaccine administration [67,71]. In the study that enrolled the greatest number of infants (1189, among which 1022 received the investigational vaccine and 167 Infanrix hexa), no significant difference in the proportion of children in whom primary immunization evoked seroprotective antibody levels was found (98.3% for Hexyon compared with 100% for Infanrix hexa) [67]. However, the number of children with antibody concentration ≥100 IU/L was higher among those treated with Infanrix hexa than in

TABLE 2. Antibody response thresholds, seroconversion rates (%) and geometric mean titres (GMT) post-primary vaccination at 2, 4 and 6 months of age and post-booster vaccination at 15–18 months of age

		Hexaxim			Infanrix hexa		
Authors	Threshold	Post-primary	Pre-booster	Post-booster	Post-primary	Pre-booster	Post-booster
Aquino *et al.* [67]	≥10 IU/L	98.3	89.8	99.4	100	95.4	100
	GMT IU/L	1142	93.3	2553	1576	127	4757
Lanata *et al.* [71]	≥10 IU/L	99.2			100		
	GMT IU/L	986			1139		
Kosalaraksa *et al.* [69]	≥10 IU/L	99.5			99.5		
	GMT IU/L	2477			2442		
Lopez *et al.* [66]	≥10 IU/L	99.7			100		
	GMT IU/L	3013			2766		

14690691, 2014, s5, Downloaded from https://onlinelibrary.wiley.com/doi/10.1111/1469-0691.12444 by Jss Medical College, Wiley Online Library on [25/07/2025]. See the Terms and Conditions (https://onlinelibrary.wiley.com/terms-and-conditions) on Wiley Online Library for rules of use; OA articles are governed by the applicable Creative Commons License

those receiving Hexyon (99.2% in comparison to 91.7%). Moreover, post-primary anti-HBs GMTs were greater in Infanrix hexa (1576 IU/L vs 1142 mIU/mL). The same findings were observed at pre-booster and post-booster doses. Similar data were collected in the second study, albeit with a significantly lower number of infants [71]. In two other studies the two hexavalent vaccines were compared in children who received it alongside PCV7 or PCV7 and rotavirus vaccine and who had received a hepatitis B vaccine at birth [66,69]. In the study by Kosalaraksa *et al.* [69] it was found that the immune response to HBsAg was similar between hexavalent vaccines and did not appear to be affected by co-administration of other vaccines. After primary series, 99.5% of children in both groups had seroprotective antibody concentration for anti-HBs of 98.4% and 99.5% in the Hexaxim and in Infantix hexa groups, respectively, had antibody levels ≥100 IU/L. No modification of immune response in comparison to historical data was found. Similar results were obtained by Lopez *et al.* [66] who found that infants given Hexyon were seroprotected after primary series in 99.7% of the cases in comparison to 100% of those who received Infanrix hexa, with no observed interference of co-administration of PCV7 and rotavirus vaccine. Similar antibody GMTs (3013 mIU/mL for the investigational vaccine and 2766 for Infanrix hexa) were detected. Persistence of seroprotection and the effect of the administration of the booster dose were evaluated in a number of studies with identical results. The study enrolling the highest number of children reported that before the booster dose seroprotection was still present in 89.8% and in 95.4% of infants included in Hexyon and Infanrix hexa groups, respectively [67]. After the booster administration these values increased to 99.4% and 100%.

The safety and tolerability observations of Hexyon derived from controlled clinical studies specifically planned to evaluate immunogenicity appear optimal. Macías *et al.* [68] found that the incidence of fever (≥39.6°C) after Hexyon was similar to that of the comparator vaccines, lower than 5%. However, the incidence of fever of any grade was lower following Hexyon than after the comparator vaccine (78.4% vs 92.7%) [68]. In general, injection-site and systemic adverse events were similarly reported between the investigational vaccine and comparators. When severe adverse events occurred, they were considered unrelated to vaccine administration, with the exception of a hypotonic–hyporesponsive episode 7 h after the first administration of Hexyon that was deemed possibly related to vaccine administration [68].

In conclusion, Hexyon was deemed to be as immunogenic as Hexavac for all antigens with the exception of HBsAg. The increase in the amount of HBsAg content and the different method used for its production seem to have resulted in higher immunogenicity. However, the data available are currently limited to the results of primary immunization and booster dose administration. No information is available regarding time persistence of seroprotection and the efficiency of immune memory. Moreover, in an era of re-emergence of pertussis, it is important to evaluate the real efficacy of a vaccine that includes only two pertussis antigens.

Conclusions

Hexavalent vaccines remain one of the best solutions to increase compliance with the recommended vaccinations for infants and children, assuring simplification of administration and costs. The major problem related to their use is their efficacy against hepatitis B. Hexavac was withdrawn from the market because it could not guarantee long-term protection against hepatitis B even in participants who were fully vaccinated during infancy, probably because of the low HBsAg content of the preparation. Infanrix hexa, which contains a double amount of HBsAg, assures a long-lasting protection and was the only hexavalent vaccine licensed for the use in children for several years. Recently, a new hexavalent vaccine, Hexaxim, has been developed and the first clinical trials have found that its immunogenicity seems to be similar to that of Infanrix hexa. However, no long-term evaluations have been performed and it is not known how for long the immune memory evoked by this preparation persists. Further studies regarding these parameters are needed. Recently collected data seem to suggest that in some cases, immune memory can wane regardless of the vaccine used for primary immunization. This suggests the need for further evaluations to define which childrens are at the highest risk and need a booster dose during late childhood to maintain protection against hepatitis B. Moreover, in comparison to Hexavac and Hexyon, Infanrix hexa has the advantage of containing three pertussis antigens (PT and FHA plus PRN). This is an additional benefit that could have an important role in the era of pertussis re-emergence.

Acknowledgements

This study was supported by a grant from the Italian Ministry of Health (Bando Giovani Ricercatori 2009).

Transparency Declaration

Susanna Esposito received research grants from Crucell, GSK, Novartis and Pfizer. Nicola Principi received a research grant

©2013 The Authors

14690691, 2014, s5, Downloaded from https://onlinelibrary.wiley.com/doi/10.1111/1469-0691.12444 by Jss Medical College, Wiley Online Library on [25/07/2025]. See the Terms and Conditions (https://onlinelibrary.wiley.com/terms-and-conditions) on Wiley Online Library for rules of use; OA articles are governed by the applicable Creative Commons License

from Pfizer. Claudia Tagliabue, Samantha Bosis, Valentina Ierardi and Monia Gambino have no conflicts of interest.

References

1. Centers for Disease Prevention and Control. Immunization Schedules. For everyone: Easy-to-read Schedules. Available at: http://www.cdc.gov/vaccines/schedules/easy-to-read/index.html (last accessed 2 August 2013).
2. Koslap-Petraco M, Judelsohn R. Societal impact of combination vaccines: experiences of physicians, nurses, and parents. *J Pediatr Health Care* 2008; 22: 300–309.
3. Madlon-Kay DJ, Harper PG. Too many shots? Parent, nurse, and physician attitudes toward multiple simultaneous childhood vaccinations. *Arch Fam Med* 1994; 3: 610–613.
4. Melman ST, Chawla T, Kaplan JM, Anbar RD. Multiple immunizations: ouch!. *Arch Fam Med* 1994; 3: 615–618.
5. Askew GL, Finelli L, Lutz J, DeGraaf J, Siegel B, Spitalny K. Beliefs and practices regarding childhood vaccination among urban pediatric providers in New Jersey. *Pediatrics* 1995; 96: 889–892.
6. Woodin KA, Rodewald LE, Humiston SG, Carges MS, Schaffer SJ, Szilagyi PG. Physician and parent opinions: are children becoming pincushions from immunizations? *Arch Pediatr Adolesc Med* 1995; 149: 845–849.
7. Zimmerman RK, Bradford BJ, Janosky JE, Mieczkowski TA, DeSensi E, Grufferman S. Barriers to measles and pertussis immunization: the knowledge and attitudes of Pennsylvania primary care physicians. *Am J Prev Med* 1997; 13: 89–97.
8. Zimmerman RK, Schlesselman JJ, Mieczkowski TA, Medsger AR, Raymund M. Physician concerns about vaccine side effects and potential litigation. *Arch Pediatr Adolesc Med* 1998; 152: 12–19.
9. Freed GL, Kauf T, Freeman VA, Pathman DE, Konrad TR. Vaccine-associated liability risk and provider immunization practices. *Arch Pediatr Adolesc Med* 1998; 152: 285–289.
10. Di Fabio JL, de Quadrosn C. Considerations for combination vaccine development and use in the developing world. *Clin Infect Dis* 2001; 33 (Suppl 4): S340–S345.
11. Centers for Disease Control and Prevention. Combination vaccines for childhood immunization recommendations of the Advisory Committee on Immunization Practices (ACIP), the American Academy of Pediatrics (AAP), and the AmericanAcademy of Family Physicians (AAFP). *MMWR* 1999; 48 (RR-5): 1–15.
12. Mallet E, Belohradsky BH, Lagos R *et al.* A liquid hexavalent combined vaccine against diphtheria, tetanus, pertussis, poliomyelitis, *Haemophilus influenzae* type b and hepatitis B: review of immunogenicity and safety. *Vaccine* 2004; 22: 1343–1357.
13. European Medicines Agency. European Medicines Agency recommends suspension of Hexavac. Available at: http://www.ema.europa.eu/ema/index.jsp?curl=pages/news_and_events/news/2009/12/news_detail_000855.jsp&mid=WC0b01ac058004d5c1 (last accessed 6 August 2013).
14. Jorgensen P, Poethko-Müller C, Hellenbrand W *et al.* Low hepatitis B immunogenicity of a hexavalent vaccine widely used in Germany: results of the German Health Survey for Children and Adolescents, 2003–2006. *Epidemiol Infect* 2010; 138: 1621–1629.
15. Agenzia Italiana del Farmaco-Istituto Superiore di Sanità- Ministero della Salute Indicazioni nazionali in seguito aalla raccomandazione EMEA sulvaccino Hexavac. Available at: htto//:wwwagenziafarnaco.it/wcss_attachment_by _id111.96280.113887100794098e.pdf?id=111.96286.1118871008131 (last accessed 6 August 2013).
16. European Medicines Agency. Scientific conclusions and grounds for the suspension of the marketing authorisation of hexavac presented by the EMEA, Available at: http://ema.europe.eu/humandocs/PDFs/EPAR/Hexavac/Hexavac-H-298-Z-28-en.pdf (last accessed 6 August 2013).
17. Olivier C, Belohradsky BH, Stojanov S, Bonnet E, Petersen G, Liese JG. Immunogenicity, reactogenicity, and safety of a seven-valent pneumococcal conjugate vaccine (PCV7) concurrently administered with a fully liquid DTPa-IPV-HBV-Hib combination vaccine in healthy infants. *Vaccine* 2008; 26: 3142–3152.
18. Swissmedic. Notification to health experts, 20 September 2005: Swissmedic suspends the license of Hexavac vaccine. *Swissmedic J* 2005; 9: 678–679.
19. Zanetti AR, Romanò L, Giambi C *et al.* Hepatitis B immune memory in children primed with hexavalent vaccines and given monovalent booster vaccines: an open-label, randomised, controlled, multicentre study. *Lancet Infect Dis* 2010; 10: 755–761.
20. Giambi C, Bella A, Barale A *et al.* A cohort study to evaluate persistence of hepatitis B immunogenicity after administration of hexavalent vaccines. *BMC Infect Dis* 2008; 8: 100.
21. Zanetti A, Parlato A, Romanò L *et al.* Challenge with a hepatitis B vaccine in two cohorts of 4–7-year-old children primed with hexavalent vaccines: an open-label, randomised trial in Italy. *Vaccine* 2012; 30: 5770–5775.
22. Schönberger K, Riedel C, Rückinger S, Mansmann U, Jilg W, Kries R. Determinants of long-term protection after hepatitis B vaccination in infancy: a meta-analysis. *Pediatr Infect Dis J* 2013; 32: 307–316.
23. Su FH, Cheng SH, Li CY *et al.* Hepatitis B seroprevalence and anamnestic response amongst Taiwanese young adults with full vaccination in infancy, 20 years subsequent to national hepatitis B vaccination. *Vaccine* 2007; 25: 8085–8090.
24. Lu CY, Ni YH, Chiang BL *et al.* Humoral and cellular immune responses to a hepatitis B vaccine booster 15–18 years after neonatal immunization. *J Infect Dis* 2008; 197: 1419–1426.
25. Romanò L, Carsetti R, Tozzi AE, Mele A, Zanetti AR. Chronic hepatitis B infection in adolescents vaccinated at birth: an alarm bell in favor of the need for a booster? *Hepatology* 2013. doi: 10.1002/hep.26458.
26. Wu Q, Zhuang GH, Wang XL, Wang LR, Li N, Zhang M. Antibody levels and immune memory 23 years after primary plasma-derived hepatitis B vaccination: results of a randomized placebo controlled trial cohort from China where endemicity is high. *Vaccine* 2011; 29: 2302–2307.
27. von Kries R, Toschke AM, Strassburger K *et al.* Sudden and unexpected deaths after the administration of hexavalent vaccines (diphtheria, tetanus, pertussis, poliomyelitis, hepatitis B, *Haemophilius influenzae* type b): is there a signal? *Eur J Pediatr* 2005; 164: 61–69.
28. Traversa G, Spila-Alegiani S, Bianchi C *et al.* Sudden unexpected deaths and vaccinations during the first two years of life in Italy: a case series study. *PLoS ONE* 2011; 6: e16363.
29. Dhillon S. DTPa-HBV-IPV/Hib vaccine (Infanrix hexa): a review of its use as primary and booster vaccination. *Drugs* 2010; 70: 1021–1058.
30. World Health Organization. WHO position paper on *Haemophilus influenzae* type b conjugate vaccines. *Wkly Epidemiol Rec* 2006; 81: 445–452.
31. Schmitt HJ, Knuf M, Ortiz E, Uwamwezi MC, Kaufhold A. Primary vaccination of infants with diphtheria-tetanus-acellular pertussis-hepatitis B virus-inactivated polio virus and *Haemophilus influenzae* type b vaccines given as either separate or mixed injections. *J Pediatr* 2000; 137: 304–312.
32. Arístegui J, Dal-Ré R, Díez-Delgado J *et al.* Comparison of the reactogenicity and immunogenicity of a combined diphtheria, tetanus, acellular pertussis, hepatitis B, inactivated polio (DTPa-HBV-IPV) vaccine, mixed with the *Haemophilus influenzae* type b (Hib) conjugate vaccine and administered as a single injection, with the DTPa-IPV/Hib and hepatitis B vaccines administered in two simultaneous injections to infants at 2, 4 and 6 months of age. *Vaccine* 2003; 21: 3593–3600.

©2013 The Authors

14690691, 2014, s5, Downloaded from https://onlinelibrary.wiley.com/doi/10.1111/1469-0691.12444 by Jss Medical College, Wiley Online Library on [25/07/2025]. See the Terms and Conditions (https://onlinelibrary.wiley.com/terms-and-conditions) on Wiley Online Library for rules of use; OA articles are governed by the applicable Creative Commons License

33. Zepp F, Knuf M, Heininger U *et al.* Safety, reactogenicity and immunogenicity of a combined hexavalent tetanus, diphtheria, acellular pertussis, hepatitis B, inactivated poliovirus vaccine and *Haemophilus influenzae* type b conjugate vaccine, for primary immunization of infants. *Vaccine* 2004; 22: 2226–2233.
34. Avdicova M, Prikazsky V, Hudeckova H, Schuerman L, Willems P. Immunogenicity and reactogenicity of a novel hexavalent DTPa-HB-V-IPV/Hib vaccine compared to separate concomitant injections of DTPa-IPV/Hib and HBV vaccines when administered according to a 3, 5 and 11 month vaccination schedule. *Eur J Pediatr* 2002; 161: 581–587.
35. Gabutti G, Zepp F, Schuerman L, Gabutti G, Zepp F, Schuerman L. Evaluation of the immunogenicity and reactogenicity of a DTPa-HB-V-IPV combination vaccine co-administered with a Hib conjugate vaccine either as a single injection of a hexavalent combination or as two separate injections at 3, 5 and 11 months of age. *Scand J Infect Dis* 2004; 36: 585–592.
36. Tichmann I, Grunert D, Habash S *et al.* Persistence of antibodies in children primed with two different hexavalent diphtheria, tetanus, acellular pertussis, hepatitis B, inactivated poliovirus and *Haemophilus influenzae* type b vaccines and evaluation of booster vaccination. *Hum Vaccin* 2006; 2: 249–254.
37. Heininger U, Sänger R, Jacquet JM, Schuerman L, DTP-HBV-IPV-059 Study Group, DTP-HBV-IPV-096 Study Group. Booster immunization with a hexavalent diphtheria, tetanus, acellular pertussis, hepatitis B, inactivated poliovirus vaccine and *Haemophilus influenzae* type b conjugate combination vaccine in the second year of life: safety, immunogenicity and persistence of antibody responses. *Vaccine* 2007; 25: 1055–1063.
38. Lim FS, Han HH, Jacquet JM, Bock HL. Primary vaccination of infants against hepatitis B can be completed using a combined hexavalent diphtheria-tetanus-acellular pertussis-hepatitis B-inactivated poliomyelitis-*Haemophilus influenzae* type B vaccine. *Ann Acad Med Singapore* 2007; 36: 801–806.
39. Pichichero ME, Blatter MM, Reisinger KS, Harrison CJ, Johnson CE, Steinhoff MC. Impact of a birth dose of hepatitis B vaccine on the reactogenicity and immunogenicity of diphtheria-tetanus-acellular pertussis-hepatitis B-inactivated poliovirus-*Haemophilus influenzae* type b combination vaccination. *Pediatr Infect Dis J* 2002; 21: 854–859.
40. Omeñaca F, Garcia-Sicilia J, García-Corbeira P *et al.* Response of preterm newborns to immunization with a hexavalent diphtheria-tetanus-acellular pertussis-hepatitis B virus-inactivated polio and *Haemophilus influenzae* type b vaccine: first experiences and solutions to a serious and sensitive issue. *Pediatrics* 2005; 116: 1292–1298.
41. Omeñaca F, Garcia-Sicilia J, Boceta R, Sistiaga-Hernando A, García-Corbeira P. Antibody persistence and booster vaccination during the second and fifth years of life in a cohort of children who were born prematurely. *Pediatr Infect Dis J* 2007; 26: 824–829.
42. Omeñaca F, Garcia-Sicilia J, García-Corbeira P, Boceta R, Torres V. Antipolyribosyl ribitol phosphate response of premature infants to primary and booster vaccination with a combined diphtheria-tetanus-acellular pertussis-hepatitis B-inactivated polio virus/*Haemophilus influenzae* type b vaccine. *Pediatrics* 2007; 119: e179–e185.
43. Tichmann-Schumann I, Soemantri P, Behre U *et al.* Immunogenicity and reactogenicity of four doses of diphtheria-tetanus-three-component acellular pertussis-hepatitis B-inactivated polio virus-*Haemophilus influenzae* type b vaccine coadministered with 7-valent pneumococcal conjugate conjugate vaccine. *Pediatr Infect Dis J* 2005; 24: 70–77.
44. Knuf M, Habermehl P, Cimino C, Petersen G, Schmitt HJ. Immunogenicity, reactogenicity and safety of a 7-valent pneumococcal conjugate vaccine (PCV7) concurrently administered with a DTPa-HBV-IPV/Hib combination vaccine in healthy infants. *Vaccine* 2006; 29: 4727–4736.
45. Knuf M, Szenborn L, Moro M *et al.* Immunogenicity of routinely used childhood vaccines when coadministered with the 10-valent pneumococcal non-typeable *Haemophilus influenzae* protein D conjugate vaccine (PHiD-CV). *Pediatr Infect Dis J* 2009; 28 (Suppl. 4): S97–S108.
46. Gimenez-Sanchez F, Kieninger DM, Kueper K *et al.* Immunogenicity of a combination vaccine containing diphtheria toxoid, tetanus toxoid, three-component acellular pertussis, hepatitis B, inactivated polio virus, and *Haemophilus influenzae* type b when given concomitantly with 13-valent pneumococcal conjugate vaccine. *Vaccine* 2011; 29: 6042–6048.
47. Whelan J, Hahné S, Berbers G, van der Klis F, Wijnands Y, Boot H. Immunogenicity of a hexavalent vaccine co-administered with 7-valent pneumococcal conjugate vaccine. Findings from the National Immunisation Programme in The Netherlands. *Hum Vaccin Immunother* 2012; 8: 743–748.
48. Tejedor JC, Moro M, Ruiz-Contreras J *et al.* Immunogenicity and reactogenicity of primary immunization with a hexavalent diphtheria-tetanus-acellular pertussis-hepatitis B-inactivated polio-*Haemophilus influenzae* type B vaccine coadministered with two doses of a meningococcal C-tetanus toxoid conjugate vaccine. *Pediatr Infect Dis J* 2006; 25: 713–720.
49. Tejedor JC, Omeñaca F, Garcia-Sicilia J *et al.* Immunogenicity and reactogenicity of a three-dose primary vaccination course with a combined diphtheria-tetanus-acellular pertussis-hepatitis B-inactivated polio-*Haemophilus influenzae* type b vaccine coadministered with a meningococcal C conjugate vaccine. *Pediatr Infect Dis J* 2004; 23: 1109–1115.
50. Knuf M, Pantazi-Chatzikonstantinou A, Pfletschinger U *et al.* An investigational tetravalent meningococcal serogroups A, C, W-135 and Y-tetanus toxoid conjugate vaccine co-administered with Infanrix™ hexa is immunogenic, with an acceptable safety profile in 12–23-month-old children. *Vaccine* 2011; 29: 4264–4273.
51. Zepp F, Behre U, Kindler K *et al.* Immunogenicity and safety of a tetravalent measles-mumps-rubella-varicella vaccine co-administered with a booster dose of a combined diphtheria-tetanus-acellular pertussis-hepatitis B-inactivated poliovirus-*Haemophilus influenzae* type b conjugate vaccine in healthy children aged 12–23 months. *Eur J Pediatr* 2007; 166: 857–864.
52. Vesikari T, Karvonen A, Prymula R *et al.* Immunogenicity and safety of the human rotavirus vaccine Rotarix co-administered with routine infant vaccines following the vaccination schedules in Europe. *Vaccine* 2010; 28: 5272–5279.
53. Zinke M, Disselhoff J, Gartner B, Jacquet JM. Immunological persistence in 4–6 and 7–9 year olds previously vaccinated in infancy with hexavalent DTPa-HBV-IPV/Hib. *Hum Vaccin* 2010; 6: 189–193.
54. Zinke M, Kappes R, Kindler K *et al.* Immune memory to hepatitis B virus in 4–9-year old children vaccinated in infancy with four doses of hexavalent DTPa-HBV-IPV/Hib vaccine. *Hum Vaccin* 2009; 5: 592–598.
55. Steiner M, Ramakrishnan G, Gartner B, Van Der Meeren O, Jacquet JM, Schuster V. Lasting immune memory against hepatitis B in children after primary immunization with 4 doses of DTPa-HBV-IPV/Hib in the first and 2nd year of life. *BMC Infect Dis* 2010; 10: 9.
56. Saenger R, Maechler G, Potreck M *et al.* Booster vaccination with hexavalent DTPa-HBV-IPV/Hib vaccine in the second year of life is as safe as concomitant DTPa-IPV/Hib + HBV administered separately. *Vaccine* 2005; 23: 1135–1143.
57. Schmitt HJ, von König CH, Neiss A *et al.* Efficacy of acellular pertussis vaccine in early childhood after household exposure. *JAMA* 1996; 275: 37–41.
58. Greco D, Salmaso S, Mastrantonio P *et al.* A controlled trial of two acellular vaccines and one whole-cell vaccine against pertussis. Progetto Pertosse Working Group. *N Engl J Med* 1996; 334: 341–348.
59. Gustafsson L, Hessel L, Storsaeter J, Olin P. Long-term follow-up of Swedish children vaccinated with acellular pertussis vaccines at 3, 5, and 12 months of age indicates the need for a booster dose at 5 to 7 years of age. *Pediatrics* 2006; 118: 978–984.

©2013 The Authors

14690691, 2014, s5, Downloaded from https://onlinelibrary.wiley.com/doi/10.1111/1469-0691.12444 by Jss Medical College, Wiley Online Library on [25/07/2025]. See the Terms and Conditions (https://onlinelibrary.wiley.com/terms-and-conditions) on Wiley Online Library for rules of use; OA articles are governed by the applicable Creative Commons License

60. Zepp F, Schmitt HJ, Cleerbout J, Verstraeten T, Schuerman L, Jacquet JM. Review of 8 years of experience with Infanrix hexa (DTPa-HBV-IPV/Hib hexavalent vaccine). *Expert Rev Vaccines* 2009; 8: 663–678.
61. McCormack PL. DTaP-IPV-Hep B-Hib vaccine (Hexaxim®): a review of its use in primary and booster vaccination. *Paediatr Drugs* 2013; 15: 59–70.
62. Nunes MC, Madhi SA. Review of a new fully liquid, hexavalent vaccine: Hexaxim. *Expert Opin Biol Ther* 2013; 13: 575–593.
63. Madhi SA, Mitha I, Cutland C, Groome M, Santos-Lima E. Immunogenicity and safety of an investigational fully liquid hexavalent combination vaccine versus licensed combination vaccines at 6, 10, and 14 weeks of age in healthy South African infants. *Pediatr Infect Dis J* 2011; 30: e68–e74.
64. Ceyhan M, Santos-Lima E. Immunogenicity and safety of an investigational hexavalent fully liquid DTaP-IPV-Hep B-PRP-T vaccine (Hexaxim®) given at 2, 3, 4 months of age and as a booster at 15–18 months compared to licensed vaccines in Turkish infants. 5th Asian Congress of Pediatric Infectious Diseases; 23-26 September 2010; Taipei, Taiwan.
65. Tregnaghi MW, Zambrano B, Santos-Lima E. Immunogenicity and safety of an investigational hexavalent diphtheria-tetanus-acellular pertussis-inactivated poliovirus-hepatitis B-*Haemophilus influenzae* B conjugate combined vaccine in healthy 2-, 4-, and 6-month-old Argentinean infants. *Pediatr Infect Dis J* 2011; 30: e88–e96.
66. Lopez P, Arguedas A, Consuelo-Miranda M. Immunogenicity and safety of a primary series of a new fully liquid DTaO-IPV-HepB-PRP-T hexavalent vaccine (Hexaxim™) co-administered with Prevenar™ and Rotarix in healthy children in America Latina. Presented at 15th International Congress of Infectious Diseases, 13–16 Lune 2012, Bangkok, Thailand.
67. Aquino AG, Brito MG, Doniz CE *et al.* A fully liquid DTaP-IPV-Hep B-PRP-T hexavalent vaccine for primary and booster vaccination of healthy Mexican children. *Vaccine* 2012; 30: 6492–6500.
68. Macías M, Lanata CF, Zambrano B *et al.* Safety and immunogenicity of an investigational fully liquid hexavalent DTaP-IPV-Hep B-PRP-T vaccine at two, four and six months of age compared with licensed vaccines in Latin America. *Pediatr Infect Dis J* 2012; 31: e126–e132.
69. Kosalaraksa P, Thisyakorn U, Benjaponpitak S, Chokephaibulkit K, Santos-Lima E. Immunogenicity and safety study of a new DTaP-IPV-Hep B-PRP-T combined vaccine compared to a licensed DTaP-IPV-Hep B//PRP-T comparator, both concomitantly administered with a 7-valent pneumococcal conjugate vaccine at 2, 4, and 6 months of age in Thai infants. *Int J Infect Dis* 2011; 15: e249–e256.
70. Madhi SA, Mitha I, Koen A. Post primary persistence and immunogeni city/safety of an investigational DRaP-IPV-Hep B-PRP-T (Hexaxim™) booster vaccine compared to licensed vaccines in South Africa. 7th Congress of the World Society for Pediatric Infectious Diseases (WSPID); 16–19 November 2011; Melbourne, Australia.
71. Lanata C, Zambrano B, Ecker L. Immunogenicity and safety of a fully liquid DTaP-IPV-Hep B-vaccine at 2–4–6 months of age in Peru. *J Vaccines* 2012; 3: 100–128.

©2013 The Authors

14690691, 2014, s5, Downloaded from https://onlinelibrary.wiley.com/doi/10.1111/1469-0691.12444 by Jss Medical College, Wiley Online Library on [25/07/2025]. See the Terms and Conditions (https://onlinelibrary.wiley.com/terms-and-conditions) on Wiley Online Library for rules of use; OA articles are governed by the applicable Creative Commons License
