# Supplementary material for: Antibody response to tetanus, diphtheria, poliomyelitis, hepatitis B, and H. influenzae b vaccines in allogeneic hematopoietic stem cell transplant adult recipients: A multicenter trial
Source: PLoS One. 2025 Oct 27;20(10):e0335224. doi: 10.1371/journal.pone.0335224 (PMC12558451; doi:10.1371/journal.pone.0335224)
Supplement: Attachment — Submitted filename: 1-s2.0-S0264410X21011464-main.pdf [file pone.0335224.s002.pdf]

Vaccine 39 (2021) 6025–6036

Contents lists available at ScienceDirect

Vaccine

journal homepage: www.elsevier.com/locate/vaccine

Review

Hexavalent vaccines: What can we learn from head-to-head studies?

Markus Knuf [a], Hervé Haas [b], Pilar Garcia-Corbeira [c], Elisa Turriani [c], Piyali Mukherjee [c], Winnie Janssens [c], Valérie Berlaimont [c,]*

[a] *Klinikum Worms, Gabriel-von-Seidl-Straße 81, 67550 Worms, Germany*
[b] *Pediatric Department, Princess Grace Hospital, Avenue Pasteur 1, 98000 Monaco, Monaco*
[c] *GSK, Avenue Fleming 20, 1300 Wavre, Belgium*

ARTICLE INFO

Article history:
Received 16 March 2021
Received in revised form 23 August 2021
Accepted 24 August 2021
Available online 13 September 2021

Keywords:
Hexavalent vaccines
Immunogenicity
Safety
Composition
Head-to-head studies

ABSTRACT

Background: Three hexavalent vaccines against diphtheria, tetanus, pertussis, poliomyelitis, hepatitis B virus (HBV), and *Haemophilus influenzae* type b (Hib) are licensed in Europe: *Infanrix hexa* (DT3aP-HBV-IPV/Hib), *Hexyon* (DT2aP-HBV-IPV-Hib) and *Vaxelis* (DT5aP-HBV-IPV-Hib).
Methods: A systematic literature search was performed in various electronic databases to identify published peer-reviewed head-to-head studies comparing any licensed hexavalent vaccine to another.
Results: Predefined inclusion criteria were met by 12 articles. Individual studies concluded that the 3 hexavalent vaccines have acceptable safety profiles although some significant differences were observed in their reactogenicity profiles. The immunogenicity of DT2aP-HBV-IPV-Hib and DT5aP-HBV-IPV-Hib was non-inferior versus DT3aP-HBV-IPV/Hib. Some differences in immune responses to common antigens were observed, but their clinical relevance was not established. Anti-filamentous hemagglutinin (FHA) from pertussis and anti-polyribosylribitol phosphate (PRP) from Hib antibody concentrations tended to be higher, and anti-HBV and anti-pertussis toxin (PT) from pertussis antibody concentrations lower in DT2aP-HBV-IPV-Hib versus DT3aP-HBV-IPV/Hib vaccinees. Anti-PT and post-primary anti-PRP antibody concentrations tended to be higher, and anti-HBV, anti-FHA, anti-pertactin from pertussis and post-booster anti-PRP antibody concentrations lower in DT5aP-HBV-IPV-Hib versus DT3aP-HBV-IPV/Hib recipients. Slightly lower immune responses towards most vaccine antigens were observed with 2 + 1 versus 3 + 1 schedules post-primary vaccination, suggesting that 2 + 1 schedules should only be considered in countries with very high vaccination coverage.
Conclusion: Although the licensed hexavalent vaccines are generally considered similar, analyses of immunogenicity data from head-to-head trials highlighted differences that could be related to differences in composition and formulation. In addition, the demonstrated non-inferiority of the immunogenicity of the more recent vaccines versus DT3aP-HBV-IPV/Hib does not allow a full bridging to similar efficacy, effectiveness and safety. The availability of DT3aP-HBV-IPV/Hib over > 20 years allowed to collect a wealth of data on its long-term immunogenicity, safety and effectiveness in clinical and post-marketing studies, and makes it a key pillar of pediatric immunization.
© 2021 GlaxoSmithKline Biologicals S.A. Published by Elsevier Ltd. This is an open access article under the CC BY license (http://creativecommons.org/licenses/by/4.0/).

Contents

1. Introduction 6026
2. Methods 6026
3. Results. 6027

Abbreviations: AE, adverse event; aP, acellular pertussis; D, diphtheria; EU, ELISA unit; FHA, filamentous hemagglutinin; FIM, Fimbriae; GMC, geometric mean concentration; GMT, geometric mean titer; HBV, hepatitis B virus; Hib, *Haemophilus influenzae* type b; IPV, inactivated poliovirus; IU, international unit; MMRV, measles-mumps-rubella-varicella vaccine; OMP, outer membrane protein; PCV13, 13-valent pneumococcal conjugate vaccine; PRN, pertactin; PRP, polyribosylribitol phosphate; PT, pertussis toxoid; SAE, serious adverse event; T, tetanus.

* Corresponding author at: GSK, Avenue Fleming 20, 1300 Wavre, Belgium.
E-mail addresses: markus.knuf@klinikum-worms.de (M. Knuf), herve.haas@chpg.mc (H. Haas), mailto:valerie.x.berlaimont@gsk.com (V. Berlaimont).

https://doi.org/10.1016/j.vaccine.2021.08.086
0264-410X/© 2021 GlaxoSmithKline Biologicals S.A. Published by Elsevier Ltd.
This is an open access article under the CC BY license (http://creativecommons.org/licenses/by/4.0/).

3.1. Composition and formulation . . . 6027
3.2. Immunogenicity in head-to-head studies . . . 6027
3.2.1. DT2aP-HBV-IPV-Hib versus DT3aP-HBV-IPV/Hib . . . 6027
3.2.2. DT5aP-HBV-IPV-Hib versus DT3aP-HBV-IPV/Hib . . . 6030
3.2.3. 3 + 1 and 2 + 1 schedules comparisons . . . 6032
3.3. Safety and reactogenicity profiles in head-to-head studies . . . 6032
3.3.1. DT2aP-HBV-IPV-Hib versus DT3aP-HBV-IPV/Hib . . . 6032
3.3.2. DT5aP-HBV-IPV-Hib versus DT3aP-HBV-IPV/Hib . . . 6033
4. Discussion . . . 6033
5. Conclusion . . . 6034
6. Trademark statement . . . 6035
Declaration of Competing Interest . . . 6035
Acknowledgements . . . 6035
Funding . . . 6035
Contributors . . . 6035
Appendix A. Supplementary material . . . 6035
References . . . 6035

1. Introduction

In infant immunization programs, combination vaccines with the greatest number of targeted diseases are hexavalent diphtheria (D), tetanus (T), pertussis, hepatitis B virus (HBV), inactivated poliovirus (IPV) and *Haemophilus influenzae* type b (Hib) vaccines. Four hexavalent acellular pertussis (aP)-containing vaccines have been licensed, of which 3 are still available on the market in Europe [1]. Two vaccines were licensed in 2000 (*Hexavac* [Sanofi Pasteur MSD S.N.C.] and *Infanrix hexa* [GSK; DT3aP-HBV-IPV/Hib]), but the marketing authorization of *Hexavac* was suspended in 2005 and withdrawn in 2012 by the European Medicines Agency due to concerns about its immunogenicity against HBV [1–3]. The third vaccine was licensed in 2012 outside the European Union and in 2013 in Europe (DT2aP-HBV-IPV-Hib, *Hexaxim*, *Hexyon* or *Hexacima*, Sanofi Pasteur) [4]. The fourth vaccine was approved in Europe in 2016 (DT5aP-HBV-IPV-Hib, *Vaxelis*, MCM Vaccine B.V.) [5].

In this review, we present differences in composition and formulation between the 3 currently licensed hexavalent vaccines [3–5]. Then, we explore the nuances of randomized head-to-head studies to critically compare their immunogenicity and safety. While studies conducted to meet registration requirements for market authorizations in different countries demonstrated the non-inferiority of immune responses induced by DT2aP-HBV-IPV-Hib or DT5aP-HBV-IPV-Hib versus DT3aP-HBV-IPV/Hib, equivalence between hexavalent vaccines was never shown.

2. Methods

A systematic literature search was performed on January 31, 2019 to identify published, peer-reviewed head-to-head studies comparing any of the 3 licensed hexavalent vaccines to another in terms of immunogenicity and safety [6]. No geographical or language restrictions were applied. A second search on PubMed, Medline, Medline in Process and Embase was performed on September 24, 2020 for more recent output. Both searches used the strategy detailed in Table S1. The reference lists of any relevant identified systematic reviews and of the European Public Assessment Reports for the 3 vaccines were also searched for studies of interest. To ensure completeness, the Trialtrove website [7] was searched to identify all head-to-head studies comparing ≥ 2 hexavalent vaccines, and completed clinical trials with published results were included. Retrieved articles were screened by 2 reviewers independently to select relevant papers based on eligibility criteria (Table S2) using a 2-step screening process (**Text S1**). Discrepancies were resolved by consensus with a 3rd researcher.

A total of 12 published articles met the predefined inclusion criteria [8–19]. Of these, 9 were identified during the initial literature search [6,8–12,14–17] and 3 during the second search [13,18,19] (Figure S1). One article on a *post-hoc* analysis in subsets of premature infants who received DT5aP-HBV-IPV-Hib or comparators in clinical trials was not further discussed because data were already analyzed in the respective studies [19].

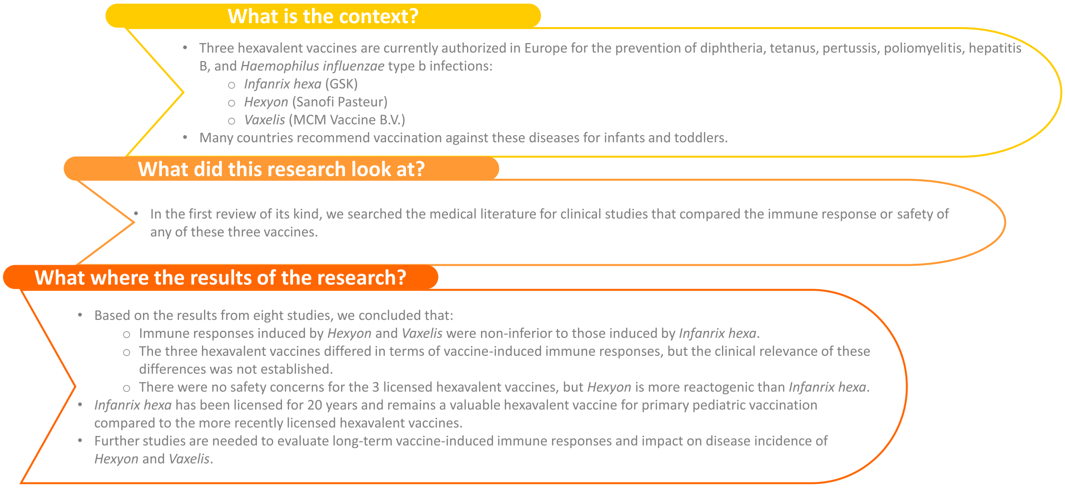

Fig. 1. Plain language summary.

A plain language summary of the findings and potential relevance and impact of this work is presented in Fig. 1.

3. Results

3.1. Composition and formulation

The 3 authorized hexavalent vaccines were developed independently, often contain the same antigens as previous lower-valency vaccines from the same manufacturer, and none are biosimilars (Table 1). The quantity of diphtheria toxoid (DT) is higher in DT3aP-HBV-IPV/Hib (≥ 30 international unit [IU], in line with the World Health Organization recommendations from 2014 [20]) compared with the other vaccines (≥ 20 IU). Other differences are the number (3 in DT3aP-HBV-IPV/Hib, 2 in DT2aP-HBV-IPV-Hib and 5 in DT5aP-HBV-IPV-Hib) and amount of pertussis antigens [21], the HBV component (recombinant *Hansenula polymorpha*-derived for DT2aP-HBV-IPV-Hib and *Saccharomyces cerevisiae*-derived for the other vaccines), the quantity of polyribosylribitol phosphate (PRP) of Hib and its carrier protein (tetanus toxoid in DT3aP-HBV-IPV/Hib and DT2aP-HBV-IPV-Hib, and outer membrane protein complex of *Neisseria meningitidis* in DT5aP-HBV-IPV-Hib) and the aluminum salts used as adjuvants. Finally, the 2 most recent vaccines have a fully-liquid formulation, while DT3aP-HBV-IPV/Hib must be reconstituted before administration by adding the suspension containing DTaP, HBV and IPV antigens from a pre-filled syringe to a vial containing the Hib lyophilized powder [22–24]. These differences in composition and formulation could be reflected in potential differences in reactogenicity, immunogenicity and/or efficacy [3–5,25].

3.2. Immunogenicity in head-to-head studies

Immune responses induced by hexavalent vaccines were evaluated by antigen-specific seroprotection rates (correlates or surrogates), vaccine response rates, geometric mean titers (GMTs) and geometric mean concentrations (GMCs) (Table S3). Established correlates of protection were used to evaluate immune responses against D, T, IPV, HBV and Hib. For pertussis antigens, there are no established correlates of protection, and immune responses were evaluated either by vaccine response rates (as defined in the protocols) or percentages of participants with ≥ 4-fold increases in antibody titers.

In head-to-head studies, non-inferiority of the immune response to a vaccine antigen was concluded if the lower limit of the 2-sided 95% confidence interval (CI) of the difference between DT2aP-HBV-IPV-Hib or DT5aP-HBV-IPV-Hib versus DT3aP-HBV-IPV/Hib in terms of seroprotection or vaccine response rate was ≥ –10 or ≥ –5 (Table S4). In descriptive comparisons, differences were detected based on non-overlapping 95% CIs.

3.2.1. DT2aP-HBV-IPV-Hib versus DT3aP-HBV-IPV/Hib

3.2.1.1. 3 + 1 schedule. The systematic literature search identified 5 studies using different immunization schedules and comparing 3-dose primary vaccination with DT2aP-HBV-IPV-Hib versus DT3aP-HBV-IPV/Hib (Table S5) [8–12]. Three studies included a booster dose administration [8,11,12]. Infants received an HBV vaccine dose in 2 studies [9,11] and Bacille Calmette-Guérin (BCG) vaccine in 1 study [11] within 1 month after birth. Long-term persistence of immune responses was evaluated for all vaccine antigens up to 4.5 years in Colombia [13] and for anti-HBV antibodies in 9–10-year-old children in Thailand [14].

3.2.1.1.1. Immune response to D. Non-inferiority of anti-D immune responses induced by 3-dose primary vaccination with DT2aP-HBV-IPV-Hib versus DT3aP-HBV-IPV/Hib was demonstrated in 2 studies (Table S4) [8,11].

In 3 studies, no differences were detected in descriptive comparisons [8,10,11]. In contrast, percentages of participants with anti-D antibody concentrations ≥ 1.0 IU/ml post-primary vaccination in Thailand (26.5% versus 12.1%) and post-booster anti-D antibody GMCs in Germany and the Czech Republic (6.08 versus 4.10 IU/mL) were higher in DT2aP-HBV-IPV-Hib versus DT3aP-HBV-IPV/Hib recipients (Table 2 and S6) [9,12].

Table 1
Characteristics and composition of the 3 hexavalent vaccines available in Europe.

Component	DT3aP-HBV-IPV/Hib	DT2aP-HBV-IPV-Hib	DT5aP-HBV-IPV-Hib
Reconstitution	Yes	No	No
Diphtheria toxoid[1,2]	≥ 30 IU	≥ 20 IU	≥ 20 IU
Tetanus Toxoid[1,2]	≥ 40 IU	≥ 40 IU	≥ 40 IU
Pertussis antigens[1,2]			
Pertussis Toxoid	25 μg	25 μg	20 μg
Filamentous Hemagglutinin	25 μg	25 μg	20 μg
Pertactin	8 μg	-	3 μg
Fimbriae type 2 and 3	-	-	5 μg
Hepatitis B surface antigen[3,4,5]	10 μg	10 μg	10 μg
Poliovirus antigens[6]			
Poliovirus type 1 (Mahoney)	40 D	40 D	40 D
Poliovirus type 2 (MEF-1)	8 D	8 D	8 D
Poliovirus type 3 (Saukett)	32 D	32 D	32 D
Hib antigens			
PRP-T[3]	10 μg/~25 μg	12 μg/22–36 μg	-
PRP-OMPC[5]	-	-	3 μg/50 μg
Adjuvant	Aluminum hydroxide, hydrated (0.50 mg Al^{3+})<br>Aluminum phosphate (0.32 mg Al^{3+})	Aluminum hydroxide, hydrated (0.60 mg Al^{3+})	Aluminum phosphate (0.17 mg Al^{3+})<br>Amorphous aluminum hydroxyphosphate sulfate (0.15 mg Al^{3+})

D, D antigen unit; Hib, *Haemophilus influenzae* type b; IU, international unit; MEF, Middle East Forces; PRP-T, polyribosylribitol phosphate conjugated to tetanus toxoid; PRP-OMPC, polyribosylribitol phosphate conjugated to outer membrane protein complex of *Neisseria meningitides*.

1 Components adsobed on hydrated aluminum hydroxide in DT3aP-HBV-IPV/Hib.

2 Components adsobed on aluminum phosphate in DT5aP-HBV-IPV-Hib.

3 Components adsobed on hydrated aluminum phosphate in DT3aP-HBV-IPV/Hib.

4 Antigen produced in yeast cells (*Saccharomyces cerevisiae* for DT3aP-HBV-IPV/Hib and DT5aP-HBV-IPV-Hib, *Hansenula polymorpha* for DT2aP-HBV-IPV-Hib.

5 Components adsobed on amorphous aluminum hydroxyphosphate sulfate in DT5aP-HBV-IPV-Hib.

6 Propagated in VERO cells for all formulations.

Table 2
Immune responses to vaccine antigens when descriptive comparisons showed a difference based on non-overlapping 95% confidence intervals between DT2aP-HBV-IPV-Hib and DT3aP-HBV-IPV/Hib after 3-dose or 2-dose primary vaccination or subsequent booster dose.

	Kosalaraksa, 2011 (2, 4, 6 months) [9]		Aquino, 2012[1,2] (2, 4, 6/15–18 months) [8]		Prymula, 2018 (2, 3, 4/11–15 months) [12]		Vesikari, 2017a (3, 5/11–12 months) [15]		2aP versus 3aP
	**2aP**	**3a**	**2aP**	**3aP**	**2aP**	**3aP**	**2aP**	**3aP**	
**HBV, post-primary (95% CI)**									
≥ 100 mIU/mL, %	–	–	91.7 (89.4; 93.7)	99.2 (95.4; 100.0)	71.9 (65.6; 77.6)	86.6 (81.5; 90.7)	–	–	<
**HBV, pre-booster (95% CI)**									
≥ 100 mIU/mL, %	–	–	–	–	44.8 (38.1; 51.6)	67.1 (60.4; 73.4)	45.2 (38.8; 51.7)	80.5 (74.9; 85.3)	<
GMC mIU/mL	–	–	–	–	74.6 (59.7; 93.2)	169 (140; 203)	76.5 (62.0; 94.4)	260 (218; 311)	<
**HBV, post-booster (95% CI)**									
≥ 100 mIU/mL, %	–	–	–	–	–	–	91.2 (86.9; 94.4)	98.0 (95.3; 99.3)	<
GMC mIU/mL	–	–	–	–	2140 (1707; 2683)	4642 (3837; 5616)	1370 (1069; 1757)	5015 (4178; 6020)	<
**D, post-primary (95% CI)**									
≥ 1.0 IU/mL, %	26.5 (20.3; 33.3)	12.1 (7.8; 17.6)	–	–	–	–	–	–	
**D, pre-booster (95% CI)**									
≥ 0.10 IU/mL, %	–	–	–	–	64.3 (57.2; 71.0)	47.8 (40.7; 54.9)	–	–	>
**D, post-booster (95% CI)**									
≥ 1.0 IU/mL, %	–	–	–	–	–	–	82.3 (76.9; 86.8)	69.9 (63.8; 75.6)	>
GMC IU/mL	–	–	–	–	6.08 (5.08; 7.27)	4.10 (3.52; 4.78)	1.70 (1.54; 1.87)	1.20 (1.07; 1.34)	>
**T, post-primary (95% CI)**									
≥ 1.0 IU/mL, %	70.9 (63.9; 77.3)	87.9 (82.4; 92.2)	–	–	–	–	–	–	<
GMC IU/mL	1.38 (1.25; 1.52)	1.83 (1.69; 1.97)	–	–	–	–	–	–	<
**P1, post-primary (95% CI)**									
GMT 1/dil	765 (649; 902)	1566 (1326; 1850)	882 (803; 970)	1370 (1082; 1736)	113 (97; 133)	268 (226; 317)	–	–	<
**P1, pre-booster (95% CI)**									
≥ 8 1/dil, %	–	–	–	–	83.7 (77.8; 88.5)	93.6 (89.3; 96.6)	62.9 (56.5; 69.0)	76.7 (70.8; 81.9)	<
GMT 1/dil	–	–	–	–	38.8 (30.8; 48.8)	80.6 (65.7; 99.0)	15.8 (12.8; 19.4)	27.3 (22.4; 33.3)	<
**P1, post-booster (95% CI)**									
GMT 1/dil	–	–	–	–	1070 (880; 1302)	2696 (2283; 3184)	1749 (1494; 2047)	3279 (2869; 3746)	<
**P2, post-primary (95% CI)**									
GMT 1/dil	1489 (1259; 1761)	2277 (1905; 2723)	1655 (1507; 1818)	2337 (1878; 2909)	191 (163; 225)	365 (305; 437)	–	–	<
**P2, pre-booster (95% CI)**									
GMT 1/dil	–	–	–	–	–	–	14.1 (11.5; 17.2)	22.4 (18.0; 27.8)	<
**P2, post-booster (95% CI)**									
GMT 1/dil	–	–	–	–	1858 (1576; 2192)	2887 (2449; 3403)	1729 (1454; 2058)	2954 (2520; 3462)	<
**P3, post-primary (95% CI)**									
GMT 1/dil	837 (695; 1007)	2029 (1646; 2502)	1106 (1005; 1218)	2186 (1752; 2727)	302 (261; 351)	662 (552; 793)	–	–	<
**P3, pre-booster (95% CI)**									
GMT 1/dil	–	–	339 (248; 463)	896 (508; 1580)	68.7 (56.6; 83.4)	135 (109;166)	–	–	<
**P3, post-booster (95% CI)**									
GMT 1/dil	–	–	6971 (5564; 8735)	13,337 (9619; 18,491)	2301 (1924; 2752)	3902 (3265; 4662)	1213 (1005; 1463)	1906 (1594; 2279)	<
**PT, post-primary (95% CI)**									
GMC EU/mL	168 (154; 183)	200 (185; 216)	–	–	–	–	–	–	<
**PT, post-booster (95% CI)**									
GMC EU/mL	–	–	–	–	112 (102; 122)	158 (144; 173)	91 (85; 97)	129 (119; 139)	<
**FHA, post-primary (95% CI)**									
≥ 4-fold increase	–	–	–	–	89.2 (84.4; 92.9)	76.4 (70.4; 81.8)	–	–	>
GMC EU/mL	148 (136; 162)	123 (113; 132)	239 (229; 250)	182 (165; 200)	–	–	–	–	>

Table 2 (*continued*)

	Kosalaraksa, 2011 (2, 4, 6 months) [9]		Aquino, 2012[1,2] (2, 4, 6/15–18 months) [8]		Prymula, 2018 (2, 3, 4/11–15 months) [12]		Vesikari, 2017a (3, 5/11–12 months) [15]		2aP versus 3aP
	2aP	3a	2aP	3aP	2aP	3aP	2aP	3aP	
**FHA, pre-booster (95% CI)**									
GMC EU/mL	–	–	–	–	34.2 (30.9; 37.9)	24.9 (22.1; 27.9)	–	–	>
**FHA, post-booster (95% CI)**									
4-fold increase	–	–	–	–	60.4 (53.5; 67.0)	80.7 (74.6; 85.9)	–	–	<
GMC EU/mL	–	–	402 (361; 446)	291 (242; 349)	–	–	–	–	>
**PRP, post-primary (95% CI)**									
≥ 0.15 µg/mL, %	–	–	–	–	–	–	71.5 (65.4; 77.2)	57.9 (51.4; 64.2)	>
≥ 1.0 µg/mL, %	85.2 (79.3; 89.9)	71.1 (64.0; 77.4)	–	–	58.9 (52.2; 65.4)	37.2 (30.9; 43.8)	–	–	>
GMC µg/mL	5.07 (4.05; 6.33)	2.41 (1.95; 2.98)	12.20 (10.80; 13.70)	6.68 (5.10; 8.74)	1.19 (0.98; 1.45)	0.60 (0.51; 0.71)	–	–	>
**PRP, pre-booster (95% CI)**									
≥ 0.15 µg/mL, %	–	–	–	–	72.0 (65.4; 77.9)	57.7 (50.7; 64.5)	–	–	>
≥ 1.0 µg/mL, %	–	–	–	–	27.1 (21.3; 33.6)	9.1 (5.6; 13.9)	–	–	>
GMC µg/mL	–	–	–	–	0.38 (0.31; 0.48)	0.17 (0.14; 0.21)	–	–	>
**PRP, post-booster (95% CI)**									
≥ 1.0 µg/mL, %	–	–	–	–	–	–	93.5 (89.6; 96.2)	85.2 (80.2; 89.4)	>
GMC µg/mL	–	–	–	–	28.8 (23.6; 35.1)	16.5 (13.6; 19.8)	9.7 (8.1; 11.7)	5.6 (4.7; 6.8)	>

2aP, DT2aP-HBV-IPV-Hib vaccine; 3aP, DT3aP-HBV-IPV/Hib vaccine; GMC, geometric mean concentration; GMT, geometric mean titer; 95% CI, 95% confidence interval; mIU/mL, milli-international units per milliliter; IU/mL, international units per milliliter; EU/mL, ELISA units per milliliter; ELISA, enzyme-linked immunosorbent assay; HBV, hepatitis B virus; T, tetanus toxoid; P1, P2 and P3, poliovirus types 1, 2 and 3; PT, pertussis toxin; D, diphtheria toxoid; PRP, polyribosylribitol phosphate; FHA, filamentous hemagglutinin.

[1] Data from 3 DT2aP-HBV-IPV-Hib vaccine lots were pooled together.

[2] DT2aP-HBV-IPV-Hib was used as the booster vaccine for all groups.

In Colombia, percentages of 4.5-year-old participants with anti-D antibody concentrations ≥ 0.1 IU/mL were 57.2% among children primed and boosted with DT2aP-HBV-IPV-Hib, 48.2% among children primed with DT2aP-HBV-IPV-Hib and boosted with DT3aP-HBV-IPV/Hib, and 59.2% among children primed with DT3aP-HBV-IPV/Hib and boosted with DT2aP-HBV-IPV-Hib [13].

3.2.1.1.2. Immune response to T. Non-inferiority of anti-T immune responses induced by 3 primary doses of DT2aP-HBV-IPV-Hib versus DT3aP-HBV-IPV/Hib was demonstrated in Colombia and Costa Rica (Table S4) [11].

No differences were detected in descriptive analyses in 3 studies [8,11,12]. In Thailand, percentages of participants with anti-T antibody concentrations ≥ 1.0 IU/ml (70.9% versus 87.9%) and anti-T antibody GMCs (1.38 versus 1.83 IU/mL) were lower post-primary vaccination for DT2aP-HBV-IPV-Hib versus DT3aP-HBV-IPV/Hib (Table 2 **and S6**) [9].

In Colombia, percentages of 4.5-year-old participants with anti-T antibody concentrations ≥ 0.1 IU/mL were 80.8% among children primed and boosted with DT2aP-HBV-IPV-Hib, 76.8% among children primed with DT2aP-HBV-IPV-Hib and boosted with DT3aP-HBV-IPV/Hib, and 81.6% among children primed with DT3aP-HBV-IPV/Hib and boosted with DT2aP-HBV-IPV-Hib [13].

3.2.1.1.3. Immune response to pertussis. Non-inferiority of immune responses to pertussis toxin (PT) and filamentous hemagglutinin (FHA) induced by 3 primary doses of DT2aP-HBV-IPV-Hib versus DT3aP-HBV-IPV/Hib was demonstrated in 2 studies (Table S4) [11,12].

No differences were detected in descriptive analyses in Colombia and Costa Rica (Table 2 and S6) [11]. Post-primary vaccination, anti-FHA antibody GMCs in Mexico (239 versus 182 enzyme-linked immunosorbent assay (ELISA) unit [EU]/mL) [8] and Thailand (148 versus 123 EU/mL) [9] and percentages of children with ≥ 4-fold increases in anti-FHA antibody concentrations in Germany and the Czech Republic (89.2% versus 76.4%) [12] were higher while anti-PT antibody GMCs in Thailand (168 versus 200 EU/mL) [9] were lower in DT2aP-HBV-IPV-Hib versus DT3aP-HBV-IPV/Hib vaccinees. Post-booster dose of DT2aP-HBV-IPV-Hib versus DT3aP-HBV-IPV/Hib, differences were observed in percentages of children with ≥ 4-fold increases in anti-FHA antibody concentrations (60.4% versus 80.7%) and anti-PT antibody GMCs (112 versus 158 EU/mL) in Germany and the Czech Republic [12]. In Mexico, differences in anti-FHA antibody GMCs (402 versus 291 EU/mL) were observed following administration of a DT2aP-HBV-IPV-Hib booster dose in children primed with DT2aP-HBV-IPV-Hib versus DT3aP-HBV-IPV/Hib [8].

In Colombia, anti-PT and anti-FHA antibody GMCs at 4.5 years of age were similar in children primed and boosted with DT2aP-HBV-IPV-Hib (3.16 and 33.8 EU/mL), primed with DT2aP-HBV-IPV-Hib and boosted with DT3aP-HBV-IPV/Hib (3.13 and 35.1 EU/mL), and primed with DT3aP-HBV-IPV/Hib and boosted with DT2aP-HBV-IPV-Hib (3.06 and 27.3 EU/mL) [13].

3.2.1.1.4. Immune response to HBV. Non-inferiority of anti-HBV immune responses induced by 3 primary doses of DT2aP-HBV-IPV-Hib versus DT3aP-HBV-IPV/Hib was demonstrated in 4 studies (Table S4) [9–12].

While no differences were detected in descriptive analyses in 3 studies (**Table 2 and S6**) [9–11], percentages of participants with post-primary vaccination anti-HBV antibody concentrations ≥ 100 mIU/mL in Germany and the Czech Republic (71.9% versus 86.6%) [12] and Mexico (91.7% versus 99.2%) [8], and post-booster anti-HBV antibody GMCs in Germany and the Czech Republic (2140 versus 4642 mIU/mL) were lower in DT2aP-HBV-IPV-Hib versus DT3aP-HBV-IPV/Hib recipients [12].

In Colombia, percentages of 4.5-year-old participants with anti-HBV antibody concentrations ≥ 10 mIU/mL were similar among children primed and boosted with DT2aP-HBV-IPV-Hib (92.3%),

primed with DT2aP-HBV-IPV-Hib and boosted with DT3aP-HBV-IPV/Hib (93.0%), and primed with DT3aP-HBV-IPV/Hib and boosted with DT2aP-HBV-IPV-Hib (94.4%) [13]. In Thailand, proportions of children with anti-HBV antibody concentrations ≥ 10 mIU/mL (49.3% and 42.9%) and anti-HBV antibody GMCs (13.3 versus 8.0 mIU/mL) were similar in 9- to 10-year-old children primed with DT2aP-HBV-IPV-Hib versus DT3aP-HBV-IPV/Hib [14]. A strong anamnestic response was observed in both groups, with post-challenge seroprotection rates of 92.8% in DT2aP-HBV-IPV-Hib-primed and 98.7% in DT3aP-HBV-IPV/Hib-primed children.

3.2.1.1.5. Immune response to poliovirus types 1, 2, 3. Non-inferiority of immune responses to poliovirus types 1, 2 and 3 induced by 3 primary doses of DT2aP-HBV-IPV-Hib versus DT3aP-HBV-IPV/Hib was demonstrated in Colombia and Costa Rica (Table S4) [11].

No differences were detected in descriptive analyses in Colombia and Costa Rica (Table 2 and S6) [11]. Post-primary vaccination, anti-poliovirus types 1, 2 and 3 antibody GMTs in Mexico (882 versus 1370; 1655 versus 2337; 1106 versus 2186) [8], Germany and the Czech Republic (113 versus 268; 191 versus 365; 302 versus 662) [12] and Thailand (765 versus 1566; 1489 versus 2277; 837 versus 2029) [9] were lower in DT2aP-HBV-IPV-Hib versus DT3aP-HBV-IPV/Hib vaccinees. Post-booster dose of DT2aP-HBV-IPV-Hib and DT3aP-HBV-IPV/Hib, differences were observed for anti-poliovirus types 1, 2 and 3 antibody GMTs (1070 versus 2696; 1858 versus 2887; 2301 versus 3902) in Germany and the Czech Republic [12]. In Mexico, differences in anti-poliovirus type 3 antibody GMTs (6971 versus 13,337) were observed following administration of a DT2aP-HBV-IPV-Hib booster dose in children primed with DT2aP-HBV-IPV-Hib versus DT3aP-HBV-IPV/Hib [8].

In Colombia, percentages of 4.5-year-old participants with anti-poliovirus types 1, 2 and 3 antibody titers ≥ 1:8 were similar among children primed and boosted with DT2aP-HBV-IPV-Hib (99.5%, 100% and 100%), primed with DT2aP-HBV-IPV-Hib and boosted with DT3aP-HBV-IPV/Hib (99.5%, 100% and 99.0%), and primed with DT3aP-HBV-IPV/Hib and boosted with DT2aP-HBV-IPV-Hib (100%, 100% and 100%) [13].

3.2.1.1.6. Immune response to Hib. Non-inferiority of anti-Hib immune responses induced by 3 primary doses of DT2aP-HBV-IPV-Hib versus DT3aP-HBV-IPV/Hib was demonstrated in 3 studies (Table S4) [9,11,12].

No differences were detected in descriptive analyses in 2 studies (Table 2 and S6) [10,11]. Post-primary vaccination, percentages of participants with anti-PRP antibody concentrations ≥ 1.0 μg/mL in Germany and the Czech Republic (58.9% versus 37.2%) [12] and Thailand (85.2% versus 71.1%) [9], and anti-PRP antibody GMCs in Mexico (12.20 versus 6.68 μg/mL) [8], Germany and the Czech Republic (1.19 versus 0.60 μg/mL) [12] and Thailand (5.07 versus 2.41 μg/mL) [9] were higher in DT2aP-HBV-IPV-Hib versus DT3aP-HBV-IPV/Hib vaccinees. Post-toddler dose, anti-PRP antibody GMCs (28.8 versus 16.5 μg/mL) were higher in DT2aP-HBV-IPV-Hib versus DT3aP-HBV-IPV/Hib recipients in Germany and the Czech Republic [12].

In Colombia, percentages of 4.5-year-old participants with anti-PRP antibody concentrations ≥ 0.15 μg/mL and ≥ 1.0 μg/mL were 100% and 85.6% among children primed and boosted with DT2aP-HBV-IPV-Hib, 100% and 84.4% among children primed with DT2aP-HBV-IPV-Hib and boosted with DT3aP-HBV-IPV/Hib, and 100% and 90.4% among children primed with DT3aP-HBV-IPV/Hib and boosted with DT2aP-HBV-IPV-Hib [13].

3.2.1.2. 2 + 1 schedule. The systematic literature search identified 1 study comparing 2 + 1 doses of DT2aP-HBV-IPV-Hib versus DT3aP-HBV-IPV/Hib administered at 3, 5 + 11–12 months of age in Sweden and Finland (Table S5) [15].

Non-inferiority of immune responses induced by DT2aP-HBV-IPV-Hib versus DT3aP-HBV-IPV/Hib was demonstrated 1 month post-primary and -booster vaccination for all common vaccine antigens, except poliovirus types 1 and 2, for which non-inferiority criteria were not met after the 2 primary doses (Table S4) [15].

While similar immune responses for some antigens were observed in descriptive analyses, potential differences were detected for others (Table 2 and S6). Post-primary vaccination, the percentage of participants with anti-PRP antibody concentrations ≥ 0.15 μg/mL was higher in DT2aP-HBV-IPV-Hib versus DT3aP-HBV-IPV/Hib vaccinees (71.5% versus 57.9%). Post-booster dose, percentages of participants with anti-D antibody concentrations ≥ 1.0 IU/mL (82.3% versus 69.9%), anti-D antibody GMCs (1.70 versus 1.20 IU/mL), percentages of participants with anti-PRP antibody concentrations ≥ 1.0 μg/mL (93.5% versus 85.2%) and anti-PRP antibody GMCs (9.7 versus 5.6 μg/mL) were higher in DT2aP-HBV-IPV-Hib versus DT3aP-HBV-IPV/Hib vaccinees. In contrast, post-booster anti-PT antibody GMCs (91 versus 129 EU/mL), percentages of participants with anti-HBV antibody concentrations ≥ 100 mIU/mL (91.2% versus 98.0%), anti-HBV antibody GMCs (1370 versus 5015 mIU/mL), and anti-poliovirus types 1, 2 and 3 antibody GMCs (1749 versus 3279; 1729 versus 2954; 1213 versus 1906) were lower in DT2aP-HBV-IPV-Hib versus DT3aP-HBV-IPV/Hib recipients.

3.2.2. DT5aP-HBV-IPV-Hib versus DT3aP-HBV-IPV/Hib

3.2.2.1. 3 + 1 schedule. The systematic literature search identified 1 study comparing 3 + 1 doses of DT5aP-HBV-IPV-Hib versus DT3aP-HBV-IPV/Hib administered at 2, 3, 4 + 12 months of age in Finland, Germany and Belgium (cc) [16]. Long-term persistence of immune responses was evaluated up to approximately 4 years post-vaccination [18].

Non-inferiority of immune responses induced by DT5aP-HBV-IPV-Hib versus DT3aP-HBV-IPV/Hib was demonstrated 1 month post-primary vaccination for D, T, poliovirus types 1, 2 and 3, and Hib antigens, and post-booster dose for pertussis and HBV antigens (Table S4) [16].

In descriptive analyses, immune responses induced by both vaccines to some antigens were similar (Table 3 and S7). However, post-booster anti-D antibody GMCs (4.73 versus 3.05 IU/mL), post-primary (0.70 versus 0.53 IU/mL) and -booster (8.16 versus 3.82 IU/mL) anti-T antibody GMCs, post-booster percentages of children with anti-T antibody concentrations ≥ 0.1 IU/mL (100.0% versus 98.0%), and post-primary (130 versus 84 EU/mL) and –booster (197 versus 91 EU/mL) anti-PT antibody GMCs were higher in DT5aP-HBV-IPV-Hib versus DT3aP-HBV-IPV/Hib vaccinees. In contrast, percentages of participants with anti-FHA vaccine response post-primary (89.0% versus 96.7%) and –booster (97.2% versus 99.8%) vaccination, anti-FHA antibody GMCs post-primary (50 versus 97 EU/mL) and -booster (122 versus 197 EU/mL) vaccination, percentages of participants with anti-PRN vaccine response (86.7% versus 92.3%) and anti-PRN antibody GMCs (46.8 versus 77.8 EU/mL) post-primary vaccination were lower in DT5aP-HBV-IPV-Hib versus DT3aP-HBV-IPV/Hib recipients. Differences were observed between DT5aP-HBV-IPV-Hib and DT3aP-HBV-IPV/Hib recipients in percentages of participants with anti-PRP antibody concentrations ≥ 0.15 μg/mL (98.4% versus 87.0%) and ≥ 1.0 μg/mL (83.3% versus 36.7%), and anti-PRP antibody GMCs (3.90 versus 0.65 μg/mL) post-primary vaccination, and anti-PRP antibody GMCs (6.79 versus 21.39 μg/mL) post-booster dose.

Approximately 4 years post-vaccination, percentages of children with anti-HBV antibody concentrations ≥ 10 mIU/mL were 70.2% in DT5aP-HBV-IPV-Hib and 82.0% in DT3aP-HBV-IPV/Hib recipients [18].

Table 3
Immune responses to vaccine antigens when descriptive comparisons showed a difference based on non-overlapping 95% confidence intervals between DT5aP-HBV-IPV-Hib and DT3aP-HBV-IPV/Hib after 3-dose or 2-dose primary vaccination and subsequent booster dose.

	**Vesikari, 2017b (2, 3, 4/12 months)** [16]		**Silfverdal, 2016 (2, 4/11–12 months)** [17]		**5aP versus 3aP**
	**5aP**	**3aP**	**5aP**	**3aP**	
**HBV, post-primary (95% CI)**					
GMC mIU/mL	–	–	226 (196; 260)	343 (288; 410)	<
**HBV, pre-booster (95% CI)**					
≥ 100 mIU/mL, %	–	–	37.8 (34.0; 41.8)	58.2 (54.1; 62.2)	<
GMC mIU/mL	81.5 (72.2; 91.9)	121.50 (107.17; 137.74)	59.5 (53.3; 66.3)	117.4 (103.3; 133.4)	<
**HBV, post-booster (95% CI)**					
GMC mIU/mL	–	–	1986 (1688; 2338)	3245 (2735; 3849)	<
**D, post-booster (95% CI)**					
≥ 0.10 IU/mL, %	–	–	83.2 (80.0; 86.2)	71.6 (67.8; 75.3)	>
GMC IU/mL	4.73 (4.32; 5.18)	3.05 (2.81; 3.32)	2.01 (1.84; 2.18)	1.46 (1.36; 1.57)	>
**T, post-primary (95% CI)**					
GMC IU/mL	0.70 (0.67; 0.73)	0.53 (0.51; 0.56)	–	–	>
**T, post-booster (95% CI)**					
≥ 0.10 IU/mL, %	100.0 (99.3; 100.0)	98.0 (96.4; 99.0)	98.8 (97.6; 99.5)	95.0 (92.9; 96.6)	>
GMC IU/mL	8.16 (7.70; 8.66)	3.82 (3.62; 4.03)	5.53 (5.21; 5.88)	3.32 (3.13; 3.53)	>
**P1, post-primary (95% CI)**					
GMT 1/dil	–	–	65 (57; 75)	88 (76; 102)	<
**P1, post-booster (95% CI)**					
GMT 1/dil	–	–	1707 (1513; 1925)	2538 (2279; 2827)	<
**P3, post-primary (95% CI)**					
GMT 1/dil	–	–	77 (66; 89)	105 (90; 123)	<
**PT, post-primary (95% CI)**					
GMC EU/mL	130 (124; 136)	84 (80; 88)	113 (107; 119)	92 (88; 97)	>
**PT, pre-booster (95% CI)**					
VR[1], %	–	–	79.4 (75.9; 82.6)	89.6 (86.9; 91.9)	<
**PT, post-booster (95% CI)**					
GMC EU/mL	197 (187; 208)	91 (86; 96)	157 (150; 166)	110 (105; 115)	>
**FHA, post-primary (95% CI)**					
VR[1], %	89.0 (86.0; 91.6)	96.7 (94.7; 98.0)	–	–	<
GMC EU/mL	50 (47; 52)	97 (92; 102)	45 (42; 47)	87 (82; 91)	<
**FHA, pre-booster (95% CI)**					
VR[1], %			58.8 (54.8; 62.7)	82.4 (79.1; 85.3)	<
GMC EU/mL	8.63 (7.99; 9.31)	22.85 (21.45; 24.34)	8.09 (7.56; 8.66)	22.70 (21.41; 24.07)	<
**FHA, post-booster (95% CI)**					
VR[1], %	97.2 (95.5; 98.4)	99.8 (99.0; 100.0)	–	–	<
GMC EU/mL	122 (116; 128)	197 (187; 207)	121 (115; 127)	204 (195; 214)	<
**PRN, post-primary (95% CI)**					
VR[1], %	86.7 (83.6; 89.5)	92.3 (89.7; 94.5)	80.3 (76.8; 83.4)	91.6 (89.0; 93.7)	<
GMC EU/mL	46.8 (42.7; 51.2)	77.8 (72.6; 83.3)	37.8 (34.1; 42.0)	78.3 (71.5; 85.7)	<
**PRN, pre-booster (95% CI)**					
GMC EU/mL	–	–	6.53 (6.02; 7.09)	11.04 (10.15; 12.02)	<
**PRN, post-booster (95% CI)**					
GMC EU/mL	–	–	104.3 (96.6; 112.5)	153.5 (143.3; 164.4)	<
**PRP, post-primary (95% CI)**					
≥ 0.15 µg/mL, %	98.4 (96.9; 99.3)	87.0 (83.8; 89.7)	96.6 (94.8; 97.9)	77.9 (74.3; 81.2)	>
≥ 1.0 µg/mL, %	83.3 (79.9; 86.3)	36.7 (32.5; 41.0)	72.9 (69.2; 76.4)	26.7 (23.2; 30.5)	>
GMC µg/mL	3.90 (3.46; 4.41)	0.65 (0.58, 0.73)	2.38 (2.13; 2.67)	0.46 (0.41; 0.51)	>
**PRP, pre-booster (95% CI)**					
≥ 0.15 µg/mL, %	94.7 (92.4; 96.4)	64.2 (59.9; 68.4)	91.4 (88.9; 93.5)	48.1 (43.9; 52.3)	>
≥ 1.0 µg/mL, %	57.8 (53.5; 62.0)	11.7 (9.1; 14.9)	50.0 (46.0; 54.2)	10.3 (8.0; 13.1)	>
GMC µg/mL	1.19 (1.07; 1.32)	0.24 (0.22; 0.26)	0.94 (0.85; 1.04)	0.17 (0.16; 0.19)	>
**PRP, post-booster (95% CI)**					
GMC µg/mL	6.79 (6.11; 7.54)	21.39 (18.77; 24.37)	4.43 (3.97; 4.94)	7.76 (6.81; 8.85)	<

5aP, DT5aP-HBV-IPV-Hib vaccine; 3aP, DT3aP-HBV-IPV/Hib; GMC, geometric mean concentration; GMT, geometric mean titer; 95% CI, 95% confidence interval; mIU/mL, milli-international units per milliliter; IU/mL, international units per milliliter; EU/mL, ELISA units per milliliter; ELISA, enzyme-linked immunosorbent assay; HBV, hepatitis B virus; T, tetanus toxoid; P1, P2 and P3, poliovirus types 1, 2 and 3; PT, pertussis toxin; D, diphtheria toxoid; PRN, pertactin; PRP, polyribosylribitol phosphate; VR, vaccine response; FHA, filamentous hemagglutinin.

[1] the VR for PT and FHA antigens was defined as follows: (1) If pre-vaccination antibody concentration was < LLOQ, then the post-vaccination antibody concentration was ≥ LLOQ, (2) If pre-vaccination antibody concentration was ≥ LLOQ, then the post-vaccination antibody concentration was ≥ pre-vaccination levels. The pre-vaccination level was defined as the antibody titer at pre-dose 1.

Table 4
Comparison of safety profiles between DT2aP-HBV-IPV-Hib versus DT3aP-HBV-IPV/Hib [6].

AE	Safety Odds Ratio of DT2aP-HBV-IPV-Hib versus DT3aP-HBV-IPV/Hib, % (95% CI)						
	Kosalaraksa, 2011[1] (2, 4, 6 months) [9]	Aquino, 2012 (2, 4, 6/15–18 months) [8]	Lanata, 2012 (2, 4, 6 months) [10]	Lopez, 2017[1,2] (2, 4, 6/12–24 months) [11]	Vesikari, 2017a[3] (3, 5/11–12 months) [15]	Prymula, 2018[3,4] (2, 3, 4/11–15 months) [12]	Overall
**Injection site, grade 3**							
Any		0.70 (0.41; 1.17)		0.62 (0.41; 0.93)	1.16 (0.76; 1.78)	0.89 (0.50; 1.59)	0.81 (0.64; 1.01)
**Injection site, any grade**							
Redness	0.87 (0.59; 1.29)	0.70 (0.50; 0.97)	0.70 (0.43; 1.14)	0.68 (0.53; 0.88)	0.69 (0.48; 1.00)	0.76 (0.54; 1.06)	0.72 (0.63; 0.83)
Pain	0.57 (0.33; 0.99)	0.54 (0.35; 0.85)	0.99 (0.56; 1.76)	0.85 (0.64; 1.14)	0.57 (0.38; 0.84)	0.92 (0.65; 1.31)	0.74 (0.62; 0.89)
Swelling	0.66 (0.44; 0.98)	0.77 (0.55; 1.07)	0.95 (0.58; 1.56)	0.94 (0.71; 1.26)	0.99 (0.70; 1.38)	0.84 (0.60; 1.20)	0.86 (0.74; 0.99)
**Systemic, grade 3**							
Any		0.76 (0.46; 1.25)		0.67 (0.48; 0.92)	0.76 (0.50; 1.16)	0.72 (0.44; 1.15)	0.71 (0.58; 0.88)
**Systemic, any grade**							
Fever[5]	0.61 (0.40; 0.92)	0.71 (0.51; 1.00)	0.97 (0.57; 1.67)	0.91 (0.70; 1.17)	0.48 (0.32; 0.72)	0.49 (0.34; 0.70)	0.67 (0.54; 0.83)
Persistent crying	0.67 (0.42; 1.08)	0.60 (0.43; 0.83)	0.78 (0.45; 1.36)	0.69 (0.52; 0.90)	0.95 (0.56; 1.60)	0.87 (0.59; 1.30)	0.72 (0.61; 0.84)
Irritability	0.89 (0.56; 1.42)	0.69 (0.48; 1.02)	0.95 (0.54; 1.66)	0.89 (0.67; 1.20)	0.46 (0.21; 1.04)	0.85 (0.56; 1.27)	0.82 (0.69; 0.98)
Drowsiness	0.70 (0.46; 1.04)	0.64 (0.45; 0.90)	1.35 (0.83; 2.21)	0.87 (0.68; 1.12)	0.78 (0.51; 1.20)	0.83 (0.57; 1.22)	0.82 (0.71; 0.94)
Anorexia	0.85 (0.58; 1.26)	0.75 (0.53; 1.06)	1.15 (0.70; 1.87)	0.76 (0.59; 0.98)	0.99 (0.69; 1.41)	0.76 (0.54; 1.06)	0.83 (0.72; 0.95)
Vomiting	1.11 (0.74; 1.65)	0.81 (0.57; 1.16)	1.15 (0.65; 2.04)	1.05 (0.82; 1.35)	1.04 (0.74; 1.47)	0.70 (0.49; 1.02)	0.96 (0.83; 1.11)

CI: confidence interval.
[1] PCV7 vaccine co-administered at primary vaccination.
[2] PCV7 vaccine co-administered at booster vaccination.
[3] PCV13 vaccine co-administered at primary vaccination.
[4] PCV13 vaccine co-administered at booster vaccination; CI, confidence interval; AE, adverse event; Overall, odds ratio calculated on pooled data [6].
[5] Body temperature grading was mostly uniform across studies (Grade 1: ≥ 38.0–38.5 °C, Grade 2: ≥ 38.6–39.5 °C, Grade 3: ≥ 39.6 °C) except in the case of Kosalaraksa, 2011 (Grade 1: ≥ 37.4–37.9 °C, Grade 2: ≥ 38.0–38.9 °C, Grade 3: ≥ 39.0 °C). PCV7, 7-valent pneumococcal conjugate vaccine; PCV13, 13-valent pneumococcal conjugate vaccine.

3.2.2.2. 2 + 1 schedule. The systematic literature search identified 1 study comparing 2 + 1 doses of DT5aP-HBV-IPV-Hib versus DT3aP-HBV-IPV/Hib administered at 2, 4 + 11–12 months of age in Sweden, Italy and Finland (Table S5) [17]. Long-term persistence of immune responses was evaluated up to approximately 4 years post-vaccination [18].

Non-inferiority of immune responses to all common antigens induced by DT5aP-HBV-IPV-Hib versus DT3aP-HBV-IPV/Hib was demonstrated 1 month post-booster dose (Table S4) [15].

While immune responses induced by both vaccines to some antigens were similar, differences were detected in descriptive analyses for others (Table 3 and S7). Percentages of children with anti-D antibody titers ≥ 0.1 IU/mL (83.2% versus 71.6%), anti-D antibody GMCs (2.01 versus 1.46 IU/mL), percentages of children with anti-T antibody titers ≥ 0.1 IU/mL (98.8% versus 95.0%) and anti-T antibody GMCs (5.53 versus 3.32 IU/mL) post-booster dose, and anti-PT antibody GMCs post-primary (113 versus 92 EU/mL) and -booster (157 versus 110 EU/mL) vaccination were higher in DT5aP-HBV-IPV-Hib versus DT3aP-HBV-IPV/Hib vaccinees. In contrast, anti-FHA antibody GMCs post-primary (45 versus 87 EU/mL) and -booster (121 versus 204 EU/mL) vaccination, anti-PRN vaccine response rates post-primary vaccination (80.3% versus 91.6%), anti-PRN antibody GMCs post-primary (37.8 versus 78.3 EU/mL) and -booster (104.3 versus 153.5 EU/mL) vaccination, anti-HBV antibody GMCs post-primary (226 versus 343 mIU/mL) and -booster (1986 versus 3245 mIU/mL) vaccination, anti-poliovirus types 1 and 3 antibody GMTs post-primary vaccination (65 versus 88 and 77 versus 105) and anti-poliovirus type 1 antibody GMTs post-toddler dose (1707 versus 2538) were lower in DT5aP-HBV-IPV-Hib versus DT3aP-HBV-IPV/Hib recipients. Differences were observed between DT5aP-HBV-IPV-Hib and DT3aP-HBV-IPV/Hib vaccinees in percentages of participants with anti-PRP antibody concentrations ≥ 0.15 μg/mL (96.6% versus 77.9%) and ≥ 1.0 μg/mL (72.9% versus 26.7%) and anti-PRP antibody GMCs (2.38 versus 0.46 μg/mL) post-primary doses, and anti-PRP antibody GMCs (4.43 versus 7.76 μg/mL) post-toddler dose (Table 3 and S7).

Approximately 4 years post-vaccination, percentages of children with anti-HBV antibody concentrations ≥ 10 mIU/mL were 65.7% in DT5aP-HBV-IPV-Hib and 83.7% in DT3aP-HBV-IPV/Hib vaccinees [18]. Anti-PT antibody GMCs (5.3 versus 3.6 EU/mL) were higher, anti-FHA antibody GMCs (6.6 versus 11.1 EU/mL) lower, and anti-PRN antibody GMCs (5.9 versus 7.2 EU/mL) similar in DT5aP-HBV-IPV-Hib versus DT3aP-HBV-IPV/Hib recipients.

3.2.3. 3 + 1 and 2 + 1 schedules comparisons

Although comparisons between studies should be interpreted cautiously, they offer valuable insight into immune responses induced by different vaccines and vaccination schedules (Tables S6 and S7). Across all vaccines, proportions of infants with seroprotective antibody levels or vaccine responses against most vaccine antigens were ≥ 84.0% and ≥ 80.3% after 3 and 2 primary doses, respectively. Lower seroprotection rates against Hib were observed in children who received 2 primary doses of DT2aP-HBV-IPV-Hib in 1 study (71.5%) and 2 primary doses of DT3aP-HBV-IPV-Hib in 2 studies (57.9% and 77.9%) [15,17]. Post-booster seroprotection or vaccine response rates were ≥ 88.9% against all antigens and were generally similar for both schedules.

3.3. Safety and reactogenicity profiles in head-to-head studies

3.3.1. DT2aP-HBV-IPV-Hib versus DT3aP-HBV-IPV/Hib

While individual studies concluded that both vaccines have acceptable safety profiles, a recent *meta*-analysis of head-to-head trials showed that redness, pain, swelling, fever, persistent crying, irritability, drowsiness and anorexia were less frequent with

Table 5
Incidence of solicited adverse events during the first 5 days post vaccination with DT5aP-HBV-IPV-Hib versus DT3aP-HBV-IPV/Hib.

	Vesikari, 2017b[1] (2, 3, 4/12 months) [16]		Silfverdal, 2016[2] (2, 4/11–12 months) [17]	
	5aP	3aP	5aP	3aP
**Injection site, any grade**				
Any	90.8	89.9	90.4	87.9
**Systemic, any grade**				
Crying	85.4	87.9	89.3	87.1
Decrease appetite	63.9	67.0	65.8	62.2
Irritability	87.9	85.7	91.6	89.4
Pyrexia	71.5	73.1	73.8	67.4
Somnolescence	76.9	80.1	86.1	80.3
Vomiting	31.8	31.0	32.8	31.0

5aP, DT5aP-HBV-IPV-Hib vaccine; 3aP, DT3aP-HBV-IPV/Hib vaccine.
[1] PCV13 and MMRV vaccines co-administered with the primary and booster hexavalent vaccine doses.
[2] PCV13 and RV1 vaccines co-administered with the primary and booster hexavalent vaccine doses. PCV13, 13-valent pneumococcal conjugate vaccine; RV1, monovalent rotavirus vaccine; MMRV, measles-mumps-rubella-varicella vaccine.

DT3aP-HBV-IPV/Hib versus DT2aP-HBV-IPV-Hib (Table 4) [6]. Grade 3 systemic reactions were less common and there was a trend towards lower frequency of grade 3 local reactions in DT3aP-HBV-IPV/Hib versus DT2aP-HBV-IPV-Hib vaccinees. The occurrence of serious adverse events (SAEs) ranged from < 2.4% [8] to 6.0% [15] in any vaccination group.

3.3.2. DT5aP-HBV-IPV-Hib versus DT3aP-HBV-IPV/Hib

In 2 head-to-head studies, similar proportions of participants reported solicited local and systemic adverse events (AEs) following administration of 3 + 1 and 2 + 1 doses of DT5aP-HBV-IPV-Hib versus DT3aP-HBV-IPV/Hib except for higher rates of pyrexia and somnolence in children who received 2 + 1 doses of DT5aP-HBV-IPV-Hib (Table 5) [16,17]. The proportions of children who reported $\geq$ 1 SAE were 2.8% and 0.8% in DT5aP-HBV-IPV-Hib recipients and 2.2% and 1.1% in DT3aP-HBV-IPV/Hib vaccinees.

4. Discussion

This critical review of head-to-head studies comparing DT2aP-HBV-IPV-Hib or DT5aP-HBV-IPV-Hib with DT3aP-HBV-IPV/Hib summarizes the observed differences between currently available hexavalent vaccines [8–12,15–17]. While the 2 more recent vaccines were registered based on studies demonstrating non-inferiority of their immunogenicity versus comparators, including DT3aP-HBV-IPV/Hib, equivalence was never demonstrated, no studies were powered to assess superiority of immune responses induced by a hexavalent vaccine versus another, and different non-inferiority thresholds were used (up to a 10% difference).

In head-to-head studies, several differences were observed in immune responses to D, T and poliovirus types 1, 2 and 3, but antibody concentrations were far above the well-defined seroprotection thresholds in all groups for these antigens [8–12,15–17]. Differences were also observed between the 3 vaccines for pertussis, PRP and HBV antigens in terms of post-primary and/or post-booster antibody concentrations, but their clinical relevance was not established [8–12,15–17]. Concerning long-term antibody persistence, safety and impact of vaccination, data are more limited for DT2aP-HBV-IPV-Hib and DT5aP-HBV-IPV-Hib, which were authorized in Europe in 2013 and 2016, than for DT3aP-HBV-IPV/Hib, which has been licensed for > 20 years and for which clinical and post-marketing evaluations are more comprehensive [3–5,25].

For pertussis, immune responses to PT seemed lower, and to FHA higher in DT2aP-HBV-IPV-Hib versus DT3aP-HBV-IPV/Hib vaccinees [8,9,11,12,15]. No head-to-head studies evaluated immune responses against PRN following DT2aP-HBV-IPV-Hib versus DT3aP-HBV-IPV/Hib vaccination since this antigen was absent from the investigational vaccine (DT2aP-HBV-IPV-Hib). The PRN protein promotes adherence of *Bordetella pertussis* to human epithelial cells. The inclusion of PRN in the vaccine leads to production of anti-PRN antibodies, which play a key role in the opsonophagocytosis of the bacteria [26–28]. In DT5aP-HBV-IPV-Hib recipients, immune responses to PT seemed higher and to PRN and FHA lower versus DT3aP-HBV-IPV/Hib recipients [16,17]. Anti-FHA antibody GMTs were close to pre-vaccination levels before the toddler dose administration in DT5aP-HBV-IPV-Hib vaccinees, suggesting a rapid waning of immunity. Of note, the presence of additional antigens in DT5aP-HBV-IPV-Hib (*Fimbriae* types 2 and 3 antigens) may offer supplementary protection against pertussis [16,17]. While immunogenicity results are difficult to interpret for pertussis in the absence of correlates of protection, the duration of protection induced by hexavalent vaccines remains an important question [26]. Persistence of anti-PT and anti-FHA antibodies was similar in 4.5 year-old children who had received 3 + 1 doses of DT2aP-HBV-IPV-Hib and DT3aP-HBV-IPV/Hib [13]. In contrast, differences observed shortly after primary vaccination in DT5aP-HBV-IPV-Hib versus DT3aP-HBV-IPV/Hib recipients, namely higher anti-PT antibody GMCs and lower anti-FHA antibody GMCs in DT5aP-HBV-IPV-Hib vaccinees, seemed to be maintained up to 4 years after completion of the 2 + 1 schedule [18]. While no studies evaluated the long-term impact beyond 4 to 5 years of the 2 most recent hexavalent vaccines, routine infant vaccination with DT3aP-HBV-IPV/Hib had a beneficial impact on pertussis incidence during 10 years in Australia [29]. Retrospective studies showed that in Italy, where DT3aP-HBV-IPV/Hib has been almost exclusively used since 2001 and vaccination coverage increased from 83.4% in 2002 to > 95% by 2011 [30,31], pertussis-related hospitalization rates progressively decreased from 1.23 to 0.64 per 100,000 inhabitants between 2001 and 2014 [32,33]. Vaccine effectiveness of DT3aP combination vaccines (both pentavalent and hexavalent vaccines) against pertussis hospitalizations was 100% (95% CI: 97.9%–100%) following a 3 + 1 vaccination course in infants in a nationwide study in Switzerland [34]. Effectiveness against pertussis of DT3aP (2 + 1 schedule) was also observed during approximately 10 years in post-marketing studies in Sweden, where DT3aP-HBV-IPV/Hib has been almost exclusively used [35,36].

For Hib, immune responses post-primary vaccination were lower with DT3aP-HBV-IPV/Hib compared with the other hexava-

lent vaccines. After the toddler dose, anti-PRP antibody concentrations seemed lower in DT3aP-HBV-IPV/Hib versus DT2aP-HBV-IPV-Hib recipients, but the opposite was observed in DT5aP-HBV-IPV-Hib vaccinees [8,9,12,15–17]. This could be explained by differential mechanisms related to the respective carrier proteins [37]. Although no trials evaluated proportions of children with anti-PRP antibody concentrations ≥ 5 μg/ml, which are potentially correlated to the prevention of colonization, post-booster anti-PRP antibody GMCs were systematically above this threshold for DT2aP-HBV-IPV-Hib and DT3aP-HBV-IPV/Hib [37,38]. While persistence of anti-PRP antibodies was similar in 4.5-year-old children who received 3 + 1 doses of DT2aP-HBV-IPV-Hib and DT3aP-HBV-IPV/Hib [13], no studies compared the persistence of anti-PRP antibodies in DT5aP-HBV-IPV-Hib versus DT3aP-HBV-IPV/Hib recipients. The estimated effectiveness of hexavalent vaccines (DT3aP-HBV-IPV/Hib and *Hexavac*) against Hib was 90.4% after 3-dose primary vaccination and 100% post-toddler dose in a nationwide surveillance German study [39]. In a case-control study in the Netherlands, vaccine effectiveness of DT3aP-HBV-IPV/Hib against Hib was 94.0% (95% CI: 89.0%–96.8%), but waned with age [40]. After hexavalent vaccine implementation into immunization programs in Italy, Hib-related hospitalization rates declined from 5.4 to 2.4 per 100,000 population among infants and from 2.3 to 0.9 per 100,000 population among 1–4-year-old children between 2001 and 2005 [31]. In Europe, the impact of Hib vaccination was sustained between 2006 and 2013, when DT3aP-HBV-IPV/Hib was almost exclusively used [41,42]. Further studies are needed to evaluate vaccine efficacy/effectiveness of the 2 more recent hexavalent vaccines against Hib.

Another difference between vaccines is the HBV component (derived from *H. polymorpha* for DT2aP-HBV-IPV-Hib and from *S. cerevisiae* for the 2 other vaccines). While in some studies lower anti-HBV antibody concentrations were induced by DT2aP-HBV-IPV-Hib and DT5aP-HBV-IPV-Hib versus DT3aP-HBV-IPV/Hib [8,12,15,17], no differences were detected in others [9–11,16]. Discrepancies in anti-HBV immune responses measured in DT3aP-HBV-IPV/Hib vaccinees may partly be explained by differences in anti-HBV assays used across head-to-head trials [43]. Indeed, the tests used in comparative studies are always directed against the antigens of the investigational vaccine (DT2aP-HBV-IPV-Hib or DT5aP-HBV-IPV-Hib in our review). Long-term anti-HBV antibody persistence was similar in 9- to 10-year-old children who received 3 primary doses of DT2aP-HBV-IPV-Hib versus DT3aP-HBV-IPV/Hib in Thailand, and strong anamnestic responses were observed in both groups [14]. Anti-HBV antibody GMCs were also similar in 4.5-year-old children who received 3 + 1 doses of DT2aP-HBV-IPV-Hib and DT3aP-HBV-IPV/Hib in Colombia [13]. In another study conducted in Finland, anti-HBV antibody GMCs were slightly lower for 6-year-old children who had received 2 + 1 doses of DT2aP-HBV-IPV-Hib versus DT3aP-HBV-IPV/Hib, but strong anamnestic responses to HBV challenges were observed for both groups (≥ 96.7%) [44]. For DT5aP-HBV-IPV-Hib, proportions of 4- to 5-year-old children with anti-HBV antibody concentrations ≥ 10 mIU/mL were significantly lower compared with DT3aP-HBV-IPV/Hib after 3 + 1 and 2 + 1 schedules, indicating a possible more rapid waning of immunity [18]. Long-term anti-HBV antibody persistence following DT3aP-HBV-IPV/Hib vaccination has also been evaluated in 14–15-year-old adolescents after a 3 + 1 schedule in Germany [45–49] and in 10–11-year-old children after a 2 + 1 schedule in Slovakia [50]. In both studies, high anamnestic responses were observed following administration of an HBV challenge and the induction of anti-HBV antibody GMCs ≥ 10 mIU/mL seemed an indicator of long-term protection. Of note, a study of the monovalent HBV vaccine containing the same HBV component as DT3aP-HBV-IPV/Hib showed antibody persistence for ≥ 20 years post-immunization [51,52]. For DT2aP-HBV-IPV-Hib (3 + 1 schedule following a birth dose of HBV vaccine), a long-term antibody persistence study in South Africa and Latin America showed that 92% of 4.5-year-old children had anti-HBV antibody concentrations ≥ 10 mIU/mL [13]. The impact of hexavalent vaccine implementation on disease incidence was shown for DT3aP-HBV-IPV/Hib (2, 4 and 6 months of age) in Australia, where the overall incidence of HBV decreased from 33.1 to 24.2 cases per 100,000 population between 2009 and 2018 [29]. Additional studies are needed to evaluate the effectiveness and long-term impact on HBV incidence of the more recent vaccines.

Concerning vaccination schedules, 2 + 1 immunization programs are increasingly used in Europe following the success of their implementation in Sweden and Italy for the last 20 years [53]. The 2 + 1 schedules decrease the cost and complexity of immunization programs but increase the importance of compliance and timeliness of vaccination and should only be considered in countries with very high vaccination coverage [30]. Slightly lower immune responses towards most antigens were observed following administration of 2 versus 3 primary doses for the 3 hexavalent vaccines, but between studies comparisons should be interpreted cautiously, and the clinical relevance of these differences, in particular for Hib, has not been established [3–5,15,54]. After the toddler dose, similar immune responses were observed for both schedules. For DT3Pa-HBV-IPV/Hib, post-marketing data from Sweden [35] and Italy [32] showed effectiveness of the 2 + 1 schedule against pertussis, and a clinical trial in Slovakia showed that this schedule induced immune memory against HBV during ≥ 10 years [50]. The immunogenicity and safety of the 2 + 1 schedule for DT2aP-HBV-IPV-Hib and DT5aP-HBV-IPV-Hib were evaluated in clinical trials, but additional effectiveness studies would be advisable to evaluate their impact on disease incidence and to ensure a successful long-term disease control.

From a public health perspective as well as for vaccinators, vaccinated children and their parents, safety is a priority and licensed vaccines must have a positive benefit/risk profile. While this was demonstrated for the 3 hexavalent vaccines, a recent meta-analysis of head-to-head trials showed a higher reactogenicity for DT2aP-HBV-IPV-Hib versus DT3aP-HBV-IPV/Hib. In this meta-analysis, the risk of experiencing 9 out of 11 analyzed solicited systemic and local adverse reactions was higher with DT2aP-HBV-IPV-Hib versus DT3aP-HBV-IPV/Hib [6], even though individual studies concluded that both vaccines have acceptable safety profiles [8–12,15]. Additional studies are needed to be able to perform meta-analyses comparing safety outcomes of DT5aP-HBV-IPV-Hib versus DT3aP-HBV-IPV/Hib or DT2aP-HBV-IPV-Hib and to conclude that the safety profiles of DT5aP-HBV-IPV-Hib and DT3aP-HBV-IPV/Hib are similar [16,17].

This is the first review to summarize comparative immunogenicity and safety data from head-to-head studies for the 3 currently licensed hexavalent vaccines. The limitations include the absence of studies comparing the 3 vaccines altogether, the different vaccines co-administered in some studies, and the different assays used to measure immune responses to some antigens. A further limitation was the different times since launch, which is not allowing the same level of evidence for the 3 vaccines. Since no systematic search was performed to identify studies evaluating the efficacy, effectiveness or impact on diseases of hexavalent vaccines, some papers may have been missed and no comparisons could be made between vaccines for these endpoints.

5. Conclusion

Although the 3 licensed hexavalent vaccines are generally considered similar, this critical review of immunogenicity and safety

data from head-to-head trials highlights that there are some differences between them, which could be related to differences in composition and formulation. The clinical relevance of the observed differences in immune responses between vaccines has not been established. Moreover, the demonstrated non-inferiority of the immunogenicity of the more recent vaccines versus DT3aP-HBV-IPV/Hib does not allow a full bridging to similar efficacy, effectiveness and safety. While the availability of DT3aP-HBV-IPV/Hib over 20 years allowed to collect a wealth of data on long-term safety, immune responses, effectiveness and impact on diseases in clinical and post-marketing studies and makes it a key pillar of pediatric immunization, data only begin to be available for DT2aP-HBV-IPV-Hib, and it is currently too early to have these data for DT5aP-HBV-IPV-Hib.

6. Trademark statement

Hexavac, *Hexaxim*, *Hexyon* and *Hexacima* are trademarks of Sanofi Pasteur. *Infanrix hexa* is a trademark owned by or licensed to the GSK group of companies. *Vaxelis* is a trademark of MCM Vaccine.

Declaration of Competing Interest

PGC is employee of the GSK group of companies. WJ, VB and ET are employees of the GSK group of companies and have shares in the GSK group of companies. MK has nothing to disclose. HH has received honoraria from GSK, Pfizer, Sanofi Pasteur, MSD, and Janssen for taking part in advisory boards and expert meetings.

Acknowledgements

Authors thank the Modis platform for editorial assistance and manuscript coordination, on behalf of GSK. Claire Verbelen provided medical writing support and Fabienne Danhier coordinated the manuscript development and provided editorial support.

Funding

This publication was funded by GlaxoSmithKline Biologicals SA, including all costs associated with the development and publication of this manuscript.

Contributors

All authors contributed to interpretation of the data and critical review of the paper for important intellectual content. All authors are in agreement with the content of the final article and have approved it for submission and publication.

Appendix A. Supplementary material

Supplementary data to this article can be found online at https://doi.org/10.1016/j.vaccine.2021.08.086.

References

[1] Dolhain J, Janssens W, Mesaros N, Hanssens L, Fierens F. Hexavalent vaccines: increasing options for policy-makers and providers. A review of the data supporting interchangeability (substitution with vaccines containing fewer antigens) and mixed schedules from the same manufacturer. Expert Rev Vacc 2018;17(6):513–24. https://doi.org/10.1080/14760584.2018.1419070.

[2] European Medicines Agency (EMA). Hexavac [diphtheria, tetanus, acellular pertussis, inactivated poliomyelitis, hepatitis b (recombinant) and haemophilus influenzae type b conjugate vaccine, adjuvanted]. Withdrawal of marketing authorisation in the European Union, https://www.ema.europa.eu/en/documents/public-statement/public-statement-hexavac-withdrawal-marketing-authorisation-european-union_en.pdf ; 2012 [accessed 05 October 2020].

[3] European Medicines Agency (EMA). Infanrix Hexa EMA summary of product characteristics, http://www.ema.europa.eu/docs/en_GB/document_library/EPAR_-_Product_Information/human/000296/WC500032505.pdf ; 2020 [accessed 05 August 2020].

[4] European Medicines Agency (EMA). Hexyon EMA summary of product characteristics, http://www.ema.europa.eu/docs/en_GB/document_library/EPAR__Product_Information/human/002796/WC500145758.pdf ; 2020 [accessed 05 August 2020].

[5] European Medicines Agency (EMA). Vaxelis EMA summary of product characteristics, http://www.ema.europa.eu/docs/en_GB/document_library/EPAR_-_Product_Information/human/003982/WC500202435.pdf ; 2020 [accessed 05 August 2020].

[6] Mukherjee P, Akpo EIH, Kuznetsova A, Knuf M, Silfverdal S-A, Kosalaraksa P, et al. Hexavalent vaccines in infants: a systematic literature review and meta-analysis of the solicited local and systemic adverse reactions of two hexavalent vaccines. Expert Rev Vaccines 2021;20:319–30. https://doi.org/10.1080/14760584.2021.1892493.

[7] Pharma Intelligence. Trialtrove: gold standard clinical trials intelligence, https://pharmaintelligence.informa.com/products-and-services/data-and-analysis/trialtrove; 2021 [accessed 11 February 2021].

[8] Aquino AGB, Brito MG, Doniz CEA, Herrera JFG, Macias M, Zambrano B, et al. A fully liquid DTaP-IPV-Hep B-PRP-T hexavalent vaccine for primary and booster vaccination of healthy Mexican children. Vaccine 2012;30(45):6492–500. https://doi.org/10.1016/j.vaccine.2012.07.040.

[9] Kosalaraksa P, Thisyakorn U, Benjaponpitak S, Chokephaibulkit K, Santos-Lima E. Immunogenicity and safety study of a new DTaP-IPV-Hep B-PRP-T combined vaccine compared to a licensed DTaP-IPV-Hep B//PRP-T comparator, both concomitantly administered with a 7-valent pneumococcal conjugate vaccine at 2, 4, and 6 months of age in Thai infants. Int J Infect Dis 2011;15(4):e249–56. https://doi.org/10.1016/j.ijid.2010.12.004.

[10] Lanata C, Zambrano B, Ecker L, Amemiya I, Gil A, Lima ES. Immunogenicity and safety of a fully liquid DTaP-IPV-Hep B-PRP-T vaccine at 2–4–6 months of Age in Peru. J Vaccines Vaccin. 2012;03. https://doi.org/10.4172/2157-7560.1000128.

[11] López P, Arguedas Mohs A, Abdelnour Vásquez A, Consuelo-Miranda M, Feroldi E, Noriega F, et al. A randomized controlled study of a fully liquid DTaP-IPV-HB-PRP-T hexavalent vaccine for primary and booster vaccinations of healthy infants and toddlers in Latin America. Pediatr Infect Dis J. 2017;36:e272-e82. http://dx.doi.org/10.1097/inf.0000000000001682.

[12] Prymula R, Kieninger D, Feroldi E, Jordanov E, B'Chir S, DaCosta X. Immunogenicity and safety of primary and booster vaccinations of a fully liquid DTaP-IPV-HB-PRP-T hexavalent vaccine in healthy infants and toddlers in Germany and the Czech Republic. Pediatr Infect Dis J 2018;37:823–30. https://doi.org/10.1097/inf.0000000000002109.

[13] Madhi SA, López P, Zambrano B, Jordanov E, B'Chir S, Noriega F, et al. Antibody persistence in pre-school children after hexavalent vaccine infant primary and booster administration. Hum Vaccin Immunother 2019;15(3):658–68. https://doi.org/10.1080/21645515.2018.1546524.

[14] Kosalaraksa P, Chokephaibulkit K, Benjaponpitak S, Pancharoen C, Chuenkitmongkol S, B'Chir S, et al. Persistence of hepatitis B immune memory until 9–10 years of age following hepatitis B vaccination at birth and DTaP-IPV-HB-PRP∼T vaccination at 2, 4 and 6 months. Hum Vaccin Immunother 2018;14(5):1257–65. https://doi.org/10.1080/21645515.2018.1426418.

[15] Vesikari T, Silfverdal SA, Jordanov E, Feroldi E. A randomized, controlled study of DTaP-IPV-HB-PRP-T, a fully liquid hexavalent vaccine, administered in a 3-, 5- and 11- to 12-month schedule. Pediatr Infect Dis J 2017;36:87–93. https://doi.org/10.1097/inf.0000000000001358.

[16] Vesikari T, Becker T, Vertruyen AF, Poschet K, Flores SA, Pagnoni MF, et al. A phase III randomized, double-blind, clinical trial of an investigational hexavalent vaccine given at two, three, four and twelve months. Pediatr Infect Dis J. 2017b;36:209-15. http://dx.doi.org/10.1097/inf.0000000000001406.

[17] Silfverdal S-A, Icardi G, Vesikari T, Flores SA, Pagnoni MF, Xu J, et al. A Phase III randomized, double-blind, clinical trial of an investigational hexavalent vaccine given at 2, 4, and 11–12 months. Vaccine 2016;34(33):3810–6. https://doi.org/10.1016/j.vaccine.2016.05.054.

[18] Vesikari T, Xu J, Johnson DR, Hall J, Marček T, Goveia MG, et al. Hepatitis B and pertussis antibodies in 4- to 5-year-old children previously vaccinated with different hexavalent vaccines. Hum Vaccin Immunother 2020;16(4):867–74. https://doi.org/10.1080/21645515.2019.1673119.

[19] Wilck MB, Xu ZJ, Stek JE, Lee AW. Safety and immunogenicity of a fully-liquid DTaP-IPV-Hib-HepB vaccine (*Vaxelis*™) in premature infants. Hum Vaccin Immunother 2021;17(1):191–6. https://doi.org/10.1080/21645515.2020.1756668.

[20] World Health Organization. Annex 4 - Recommendations to assure the quality, safety and efficacy of diphtheria vaccines (adsorbed). Replacement of Annex 2 of WHO Technical Report Series, No. 800, and Annex 5 of WHO Technical Report Series, No. 927, https://www.who.int/biologicals/vaccines/Diphtheria_Recommendations_TRS_980_Annex_4.pdf?ua=1 ; 2014 [accessed 05 October 2020].

[21] Harriman K, Healy CM. How important is the type of acellular pertussis vaccine? Clin Infect Dis 2019;70:208–9. https://doi.org/10.1093/cid/ciz205.

[22] Kumar G, Padhiar A, Carroll S, Woods B, Shread K. Estimating the cost impact of switching from a vial to a pre-filled syringe mode of administration for the DTaA-IPV-Hib '5-In-1' vaccine in infants. Value Health 2013;16(7):A345. https://doi.org/10.1016/j.jval.2013.08.135.

[23] Wiedenmayer KA, Weiss S, Chattopadhyay C, Mukherjee A, Kundu R, Ayé R, et al. Simplifying paediatric immunization with a fully liquid DTP–HepB–Hib combination vaccine: evidence from a comparative time-motion study in India. Vaccine 2009;27(5):655–9. https://doi.org/10.1016/j.vaccine.2008.11.045.

[24] De Coster I, Fournie X, Faure C, Ziani E, Nicolas L, Soubeyrand B, et al. Assessment of preparation time with fully-liquid versus non-fully liquid paediatric hexavalent vaccines. A time and motion study. Vaccine 2015;33(32):3976–82. https://doi.org/10.1016/j.vaccine.2015.06.030.

[25] Puente Gómez I, Verheust C, Hanssens L, Dolhain J. Safety profile of *Infanrix hexa* – 17 years of GSK's passive post-marketing surveillance. Expert Rev Vacc 2020;19(8):771–9. https://doi.org/10.1080/14760584.2020.1800458.

[26] Poolman JT, Hallander HO. Acellular pertussis vaccines and the role of pertactin and fimbriae. Expert Rev Vacc 2007;6(1):47–56. https://doi.org/10.1586/14760584.6.1.47.

[27] Leininger E, Roberts M, Kenimer JG, Charles IG, Fairweather N, Novotny P, et al. Pertactin, an Arg-Gly-Asp-containing *Bordetella pertussis* surface protein that promotes adherence of mammalian cells. Proc Natl Acad Sci USA 1991;88(2):345–9. https://doi.org/10.1073/pnas.88.2.345.

[28] Hellwig SM, Rodriguez M, Berbers GM, van de Winkel JJ, Mooi F. Crucial role of antibodies to pertactin in *Bordetella pertussis* immunity. J Infect Dis 2003;188(5):738–42. https://doi.org/10.1086/jid.2003.188.issue-510.1086/377283.

[29] Bayliss J, Nissen M, Prakash D, Richmond P, Oh K-B, Nolan T. Control of vaccine preventable diseases in Australian infants: reviewing a decade of experience with DTPa-HBV-IPV/Hib vaccine. Hum Vaccin Immunother 2021;17(1):176–90. https://doi.org/10.1080/21645515.2020.1764826.

[30] Baldo V, Bonanni P, Castro M, Gabutti G, Franco E, Marchetti F, et al. Combined hexavalent diphtheria-tetanus-acellular pertussis-hepatitis B-inactivated poliovirus-*Haemophilus influenzae* type b vaccine; *Infanrix*™ *hexa*. Hum Vaccin Immunother 2014;10(1):129–37. https://doi.org/10.4161/hv.26269.

[31] Martinelli D, Azzari C, Bonanni P, Esposito S, Franco E, Icardi G, et al. Impact of *Haemophilus influenzae* type b conjugate vaccination on hospitalization for invasive disease in children fifteen years after its introduction in Italy. Vaccine 2017;35(46):6297–301. https://doi.org/10.1016/j.vaccine.2017.09.077.

[32] Gabutti G, Rota MC, Bonato B, Pirani R, Turlà G, Cucchi A, et al. Hospitalizations for pertussis in Italy, 1999–2009: analysis of the hospital discharge database. Eur J Pediatr 2012;171(11):1651–5. https://doi.org/10.1007/s00431-012-1791-8.

[33] Brosio F, Kuhdari P, Cocchio S, Stefanati A, Baldo V, Gabutti G. Impact of pertussis on the Italian population: Analysis of hospital discharge records in the period 2001–2014. Int J Infect Dis 2020;91:143–8. https://doi.org/10.1016/j.ijid.2019.10.027.

[34] Mack I, Erlanger TE, Lang P, Sinniger P, Perisa D, Heininger U. Dose-dependent effectiveness of acellular pertussis vaccine in infants: a population-based case-control study. Vaccine 2020;38(6):1444–9. https://doi.org/10.1016/j.vaccine.2019.11.069.

[35] Hallander HO, Gustafsson L. Efficacy and effectiveness of acellular pertussis vaccines: a 20-year Swedish experience. Expert Rev Vacc 2009;8(10):1303–7. https://doi.org/10.1586/erv.09.88.

[36] Aronsson B, Källberg H, Byström E, Storsaeter J, Roth A, Andersson S. Vaccine effectiveness of acellular pertussis vaccine estimated by the screening method, Swedish preschool children, 2007-2018 [abstract]. In Proceedings of the 38th Annual Meeting of The European Society for Paediatric Infectious Diseases (ESPID); 2020 October 26-29; Virtual Meeting; Abstract O071.

[37] Slack M, Esposito S, Haas H, Mihalyi A, Nissen M, Mukherjee P, et al. *Haemophilus influenzae* type b disease in the era of conjugate vaccines: critical factors for successful eradication. Expert Rev Vac 2020;19(10):903–17. https://doi.org/10.1080/14760584.2020.1825948.

[38] Fernandez J, Levine O, Sanchez J, Balter S, LaClaire L, Feris J, et al. Prevention of *Haemophilus influenzae* type b colonization by vaccination: correlation with serum anti-capsular IgG concentration. J Infect Dis 2000;182(5):1553–6. https://doi.org/10.1086/jid.2000.182.issue-510.1086/315870.

[39] Kalies H, Grote V, Siedler A, Gröndahl B, Schmitt H-J, von Kries R. Effectiveness of hexavalent vaccines against invasive *Haemophilus influenzae* type b disease: Germany's experience after 5 years of licensure. Vaccine 2008;26(20):2545–52. https://doi.org/10.1016/j.vaccine.2008.03.001.

[40] Monge S, Hahné SJM, de Melker HE, Sanders EAM, van der Ende A, Knol MJ. Effectiveness of the DTPa-HBV-IPV/Hib vaccine against invasive *Haemophilus influenzae* type b disease in the Netherlands (2003–16): a case-control study. Lancet Infect Dis 2018;18(7):749–57. https://doi.org/10.1016/S1473-3099(18)30166-X.

[41] Wang S, Tafalla M, Hanssens L, Dolhain J. A review of *Haemophilus influenzae* disease in Europe from 2000–2014: challenges, successes and the contribution of hexavalent combination vaccines. Expert Rev Vacc 2017;16(11):1095–105. https://doi.org/10.1080/14760584.2017.1383157.

[42] Robert Koch-Institut. Epidemiologisches Bulletin 26 / 2020. 2020. http://dx.doi.org/10.25646/7072.

[43] Avdicová M, Prikazský V, Hudecková H, Schuerman L, Willems P. Immunogenicity and reactogenicity of a novel hexavalent DTPa-HBV-IPV/Hib vaccine compared to separate concomitant injections of DTPa-IPV/Hib and HBV vaccines, when administered according to a 3, 5 and 11 month vaccination schedule. Eur J Pediatr 2002;161(11):581–7. https://doi.org/10.1007/s00431-002-1079-5.

[44] Virta M, Soininen A, Patel DM, Petit C, Tabar C, Lyabis O. Persistence of hepatitis B immune memory until 6 years of age following hexavalent DTaP-IPV-HB-PRP∼T vaccination in a 3-, 5- and 11- to 12-month schedule and response to a subsequent hepatitis B challenge vaccination. Pediatr Infect Dis J 2021;40:e28–30. https://doi.org/10.1097/inf.0000000000002954.

[45] Schwarz TF, Behre U, Adelt T, Donner M, Suryakiran PV, Janssens W, et al. Long-term antibody persistence against hepatitis B in adolescents 14–15-years of age vaccinated with 4 doses of hexavalent DTPa-HBV-IPV/Hib vaccine in infancy. Hum Vaccin Immunother 2019;15(1):235–41. https://doi.org/10.1080/21645515.2018.1509658.

[46] Behre U, Van Der Meeren O, Crasta P, Hanssens L, Mesaros N. Lasting immune memory against hepatitis B in 12–13-year-old adolescents previously vaccinated with 4 doses of hexavalent DTPa-HBV-IPV/Hib vaccine in infancy. Hum Vaccin Immunother 2016;12(11):2916–20. https://doi.org/10.1080/21645515.2016.1202388.

[47] Zinke M, Disselhoff J, Gartner B, Marie Jacquet J. Immunological persistence in 4–6 and 7–9 year olds previously vaccinated in infancy with hexavalent DTPa-HBV-IPV/Hib. Hum Vaccin 2010;6(2):189–93. https://doi.org/10.4161/hv.6.2.10117.

[48] Steiner M, Ramakrishnan G, Gartner B, Van Der Meeren O, Jacquet JM, Schuster V. Lasting immune memory against hepatitis B in children after primary immunization with 4 doses of DTPa-HBV-IPV/Hib in the first and 2nd year of life. BMC Infect Dis 2010;10:9. https://doi.org/10.1186/1471-2334-10-9.

[49] Van Der Meeren O, Bleckmann G, Crasta PD. Immune memory to hepatitis B persists in children aged 7–8 years, who were vaccinated in infancy with 4 doses of hexavalent DTPa-HBV-IPV/Hib (*Infanrix*™ *hexa*) vaccine. Hum Vaccin Immunother 2014;10(6):1682–7. https://doi.org/10.4161/hv.28480.

[50] Avdicova M, Crasta PD, Hardt K, Kovac M. Lasting immune memory against hepatitis B following challenge 10–11 years after primary vaccination with either three doses of hexavalent DTPa-HBV-IPV/Hib or monovalent hepatitis B vaccine at 3, 5 and 11–12 months of age. Vaccine 2015;33(23):2727–33. https://doi.org/10.1016/j.vaccine.2014.06.070.

[51] Poovorawan Y, Chongsrisawat V, Theamboonlers A, Leroux-Roels G, Kuriyakose S, Leyssen M, et al. Evidence of protection against clinical and chronic hepatitis B infection 20 years after infant vaccination in a high endemicity region. J Viral Hepat 2011;18:369–75. https://doi.org/10.1111/j.1365-2893.2010.01312.x.

[52] Van Damme P, Dionne M, Leroux-Roels G, Van Der Meeren O, Di Paolo E, Salaun B, et al. Persistence of HBsAg-specific antibodies and immune memory two to three decades after hepatitis B vaccination in adults. J Viral Hepat 2019;26:1066–75. https://doi.org/10.1111/jvh.13125.

[53] European Medicines Agency (EMA). Vaccine schedules in all countries of the European Union, https://vaccine-schedule.ecdc.europa.eu/ ; 2021 [accessed 16 February 2021].

[54] Syed YY. DTaP5-HB-IPV-Hib vaccine (*Vaxelis*®): a review of its use in primary and booster vaccination. Paediatr Drugs 2017;19(1):69–80. https://doi.org/10.1007/s40272-016-0208-y.
