# Supplementary material for: Antibody response to tetanus, diphtheria, poliomyelitis, hepatitis B, and H. influenzae b vaccines in allogeneic hematopoietic stem cell transplant adult recipients: A multicenter trial
Source: PLoS One. 2025 Oct 27;20(10):e0335224. doi: 10.1371/journal.pone.0335224 (PMC12558451; doi:10.1371/journal.pone.0335224)
Supplement: Attachment — Submitted filename: Response to editorial comments.docx [file pone.0335224.s004.docx]

**Manuscript PONE-D-25-32490**

*Antibody response to tetanus, diphtheria, poliomyelitis, hepatitis B, and H. influenzae b vaccines in allogeneic hematopoietic stem cell transplant adult recipients: A multicenter trial*

**Response to editorial comments**

**1. Please ensure that your manuscript meets PLOS ONE's style requirements, including those for file naming. The PLOS ONE style templates can be found at https://journals.plos.org/plosone/s/file?id=wjVg/PLOSOne_formatting_sample_main_body.pdf and https://journals.plos.org/plosone/s/file?id=ba62/PLOSOne_formatting_sample_title_authors_affiliations.pdf**

We ensured this.

**2. We note that you have indicated that there are restrictions to data sharing for this study. For studies involving human research participant data or other sensitive data, we encourage authors to share de-identified or anonymized data. However, when data cannot be publicly shared for ethical reasons, we allow authors to make their data sets available upon request. For information on unacceptable data access restrictions, please see** [**http://journals.plos.org/plosone/s/data-availability#loc-unacceptable-data-access-restrictions**](http://journals.plos.org/plosone/s/data-availability#loc-unacceptable-data-access-restrictions)**.**

**Before we proceed with your manuscript, please address the following prompts:**

**a) If there are ethical or legal restrictions on sharing a de-identified data set, please explain them in detail (e.g., data contain potentially identifying or sensitive patient information, data are owned by a third-party organization, etc.) and who has imposed them (e.g., a Research Ethics Committee or Institutional Review Board, etc.). Please also provide contact information for a data access committee, ethics committee, or other institutional body to which data requests may be sent.**

We created a totally de-identified set of data and deposit it on the OST (Center for Open Science) repository, with the DOI 10.17605/OSF.IO/KYDU6 on the OSF repository (osf.io).

**b) If there are no restrictions, please upload the minimal anonymized data set necessary to replicate your study findings to a stable, public repository and provide us with the relevant URLs, DOIs, or accession numbers. Please see http://www.bmj.com/content/340/bmj.c181.long for guidelines on how to de-identify and prepare clinical data for publication. For a list of recommended repositories, please see https://journals.plos.org/plosone/s/recommended-repositories. You also have the option of uploading the data as Supporting Information files, but we would recommend depositing data directly to a data repository if possible.**

**Please update your Data Availability statement in the submission form accordingly.**

We added this statement at the end of the manuscript.

**3. We note that the grant information you provided in the ‘Funding Information’ and ‘Financial Disclosure’ sections do not match.**

We harmonized the way things were presented in the manuscript and on the submission website.

**When you resubmit, please ensure that you provide the correct grant numbers for the awards you received for your study in the ‘Funding Information’ section.**

We ensured this.

**4. Thank you for stating the following financial disclosure:**

**This study was funded by the national French hospital research program (PHRC) (Grant N° PHRCI-16-016).**

**Please state what role the funders took in the study. If the funders had no role, please state:** **"The funders had no role in study design, data collection and analysis, decision to publish, or preparation of the manuscript." If this statement is not correct you must amend it as needed.**

**Please include this amended Role of Funder statement in your cover letter; we will change the online submission form on your behalf.**

This statement is correct. We added this sentence in the revised version of the manuscript.

**5. Please note that funding information should not appear in any section or other areas of your manuscript. We will only publish funding information present in the Funding Statement section of the online submission form. Please remove any funding-related text from the manuscript.**

We removed the other funding-related text in the revised version of the manuscript.

**6. If the reviewer comments include a recommendation to cite specific previously published works, please review and evaluate these publications to determine whether they are relevant and should be cited. There is no requirement to cite these works unless the editor has indicated otherwise.**

We did not cite new articles in the revised version of the manuscript.

**7. Please review your reference list to ensure that it is complete and correct. If you have cited papers that have been retracted, please include the rationale for doing so in the manuscript text, or remove these references and replace them with relevant current references. Any changes to the reference list should be mentioned in the rebuttal letter that accompanies your revised manuscript. If you need to cite a retracted article, indicate the article’s retracted status in the References list and also include a citation and full reference for the retraction notice.**

We performed this review. Particularly, no retracted articles were cited.

**Response to reviewers' comments**

**Reviewer #1**

**The manuscript #PONE-D-25-32490 titled “Antibody response to tetanus, diphtheria, poliomyelitis, hepatitis B, and H. influenzae b vaccines in allogeneic hematopoietic stem cell transplant adult recipients: A multicenter trial” described antibody response to tetanus, diphtheria, hepatitis B, polio and H. influenzae b after 4 doses of DTaP-HBV-Hib-IPV in adults after hematopoietic stem cell transplant. The study was interesting. I have the following comments,**

**1. In my opinion, a lot of studies have demonstrated good response of immune response after vaccination in patients after HSCT. The authors may have raise the novel issues this study contributed to.**

Only few studies assessed the antibody response to 5 pathogens/diseases vaccines in adult HSCT recipients (1 when this study was designed, and 3 when the manuscript was submitted to PLOS One). We added a sentence in the discussion to enlighten this novelty.

**2. In result part, the percentages of patients having protected antibody seemed to decrease in tetanus, diphtheria, polio. What would be the explanation of these results?**

The decrease is very low, or non-existent, between months 3 and 13 (tetanus: 97.2% and 97.5%, diphtheria: 100% and 97.5%, polio: 96.6% and 92.7%). It may be debatable to speculate on such a small difference.

**In addition, why were the percentages of patients not protected for pathogens higher after the booster dose compared with after the 3rd dose? In fact, these percentages should be lower after the booster dose. Did some of these patients get additional immunosuppressive drugs during the vaccination course?**

For this parameter too, the differences are very limited (and the proportion of non-protected are low, less than 5%), and may reflect the non-protection of a single one participant; we are maybe not sure that we should comment in the “Discussion” section such tiny variations.

**3. Did the presence of GvHD affect immune response to vaccination?**

As we excluded patients with a current GvHD, we did not have this data. However, we did not analyze the impact of a past GvHD. This has would indeed be relevant; it is a limitation, and we noted this in the “Discussion” section of the revised manuscript.

**4. Concomitant vaccinations should be presented.**

This indeed important. We do not have the data for each patient. For example, anti-meningococcal vaccination (B vaccine, conjugated polysaccharide tetravalent ACWY vaccine) is generally initiated alongside with the hexavalent vaccination; according to the season, influenza vaccine could be also co-administered; Covid-19 vaccine boost may have also been co-administered. We add this point in the revised version of the manuscript.

**5. Minor grammatical correction should be performed.**

Thank you for this remark. We performed these corrections.

**Reviewer #2**

**Considering that the 2 vaccines used have different formulations (DTap2 vs DTap3) and that the immunogenicity values differ in both, should they have been analyzed independently in order to decide withether DTaP 3 is better or not (Hexavalent vaccines: What can we learn from head-to-head studies?**

**Markus Knuf , Hervé Haas, Pilar Garcia-Corbeira , Elisa Turriani , Piyali Mukherjee ,Winnie Janssens , Valérie Berlaimont Vaccine Volume 39, Issue 41, 1 October 2021, Pages 6025-6036)(Hexavalent CMIvaccines for immunization in paediatric age S. Esposito, C. Tagliabue, S. Bosis, V. Ierardi, M. Gambino and N. Principi Clinical Microbiology and Infection, Volume 20 Supplement 5, May 2014 76-85**

The reviewer probably refers to names such as DT3aP-HBV-IPV/Hib used in the cited GSK article (we did no use tetravalent vaccines (DTap) but only hexavalent vaccines).

Indeed, several hexavalent vaccines were used during the study, as we did not restrict the study to one vaccine manufacturer (this would have led to increase too much the study costs, considering the funding received). This heterogeneity may have led to difference in vaccine immunogenicity, but in a way almost impossible to assess, as different vaccines may have been used in the same patient. However, this heterogeneity reflects the “real world” immunization practice, where hospital pharmacy may change from one year to another the manufacturer for a vaccine. We acknowledged this important point in the revised version of the manuscript (it was already acknowledged [“*the 2 vaccine combinations we used (Infanric hexa® and Hexyon®) are no totally identical in antigen quantities of* Haemophilus influenzae *type b polysaccharide, of diphtheria toxoid, of inactivated poliomyelitis virus”*], but we stressed more this point).

**Reviewer #3**

**Useful research and give good protection to the transplant patients . Excellent efforts .and choosing these type of research is really needed to the dither generation . Transplant patient survival and morbidity free survival is very important and for that this research is very useful .**

Thank you for this comment.
