# Supplementary material for: Antibody response to tetanus, diphtheria, poliomyelitis, hepatitis B, and H. influenzae b vaccines in allogeneic hematopoietic stem cell transplant adult recipients: A multicenter trial
Source: PLoS One. 2025 Oct 27;20(10):e0335224. doi: 10.1371/journal.pone.0335224 (PMC12558451; doi:10.1371/journal.pone.0335224)
Supplement: Attachment — Submitted filename: Response to reviewer comments - R2.docx [file pone.0335224.s005.docx]

PONE-D-25-32490R1

*Antibody response to tetanus, diphtheria, poliomyelitis,*

*hepatitis B, and H. influenzae b vaccines*

*in allogeneic hematopoietic stem cell transplant adult recipients: A multicenter trial*

**Responses to Review Comments to the Author (Revision N°2)**

**Reviewer #1**

**The manuscript #PONE-D-25-32490R1 titled “Antibody response to tetanus, diphtheria, poliomyelitis, hepatitis B, and H. influenzae b vaccines in allogeneic hematopoietic stem cell transplant adult recipients: A multicenter trial” described antibody response to tetanus, diphtheria, hepatitis B, polio and H. influenzae b after 4 doses of DTaP-HBV-Hib-IPV in adults after hematopoietic stem cell transplant. In my opinion, the previous issues were not quite well addressed.**

1. **In my opinion, no novel issues were pointed out from the existing knowledge. At least, please consider this reviewed article; doi: 10.3390/cancers13236140.**

This comment is a bit surprising, as we are currently at the “revision” part of the process; meanwhile, this remark is understandable in the “initial submission” part of the process, not in the revision version (indeed, the data we reported in the revised version are not more or less “novel” than in the initial submission of the manuscript).

Moreover, as mentioned in the manuscript, this clinical trial brings immunogenicity data regarding the vaccine response in a rarely studied population: adult HSCT recipients. Only few studies reported such data (response to diphtheria, tetanus, polio vaccines: 4 studies, all smaller than ours; response to *Haemophilus*: 5 studies, all smaller than ours; response to hepatitis B in non-infected subjects: 2 studies, one being smaller than ours) in these subjects.

However, we modified again the R2 version of the manuscript to make this clearer, and added citations concerning small (N<30) studies.

Regarding the suggesting citation: the title is very interesting and promising, but we usually do not cite predatory editors, and therefore we prefer not to cite manuscripts published by MDPI journals (<https://www.predatoryjournals.org/news/is-mdpi-predatory>; <https://en.wikipedia.org/wiki/MDPI#Controversies>), as giving publicity to such problematic stakeholders may be considered malpractice.

2. **In result part, the percentages of patients having protected antibody seemed to decrease in tetanus, diphtheria, polio. What would be the explanation of these results? This should be better addressed.**

We had stated in the “response to reviewers” document during the R1 revision process that “*The decrease is very small, or non-existent, between months 3 and 13 (tetanus: 97.2% to 97.5%, diphtheria: 100% to 97.5%, polio: 96.6% to 92.7%). It may be debatable to speculate on such a small difference*”.

3. **Did the presence of GvHD affect immune response to vaccination? Previous GvHD could affect later immune response as immune reconstitution may be delayed.**

This is indeed an important remark. We had already stated in the “response to reviewers” document that “*As we excluded patients with a current GvHD, we did not have this data. However, we did not analyze the impact of a past GvHD. This has would indeed be relevant; it is a limitation, and we noted this in the “Discussion” section of the revised manuscript*”.

On the other hand, we previously did not exploit the data available to us concerning the presence of non-severe GvHD at inclusion. We therefore did these analyses in the recent days and did not observe any difference between those with or without GvHD at inclusion. We added this result in the revised (R2) manuscript.

**Addressing only CD19+ level and immunoglobulin level are not enough. T cell subset numbers or functions are also important in immune response.**

This is an important remark, and indeed, we also measured TCD3, 4 and 8 lymphocytes populations in the participants, alongside with CD19. As stated since the initial submission of the manuscript, “*For all vaccines, we observed that the antibody level at M13 did not significantly differ according to age, sex, type of hematological malignancy, delay between transplantation and vaccination, type of transplant conditioning, or (except when mentioned below) blood levels of gammaglobulins, CD4+ T lymphocytes, or CD8+ T lymphocytes at inclusion. The only association that emerged from multivariate linear regression linked the anti-diphtheria antibody level to CD8+ T lymphocytes and the anti-H. influenzae b antibody level to both gammaglobulins and CD8+ T lymphocytes*”.

4. **Concomitant vaccinations should be presented. Interference of other vaccines may affect immune response. This should be clearly presented.**

We had stated in the “response to reviewers” document during the R1 revision process that “*This is indeed important. [However, ] we do not have the data for each patient. For example, anti-meningococcal vaccination (B vaccine, conjugated polysaccharide tetravalent ACWY vaccine) is generally initiated alongside with the hexavalent vaccination; according to the season, influenza vaccine could be also co-administered; Covid-19 vaccine boost may have also been co-administered. We add this point in the revised version of the manuscript*.”

We still thing that this comment is very relevant, but we still do not have the data. We have let in the revised (R2) version of the manuscript the sentence we had already introduced in the R1 revised version to acknowledge this (“*We did not collect if (and which) other vaccines (e.g., meningococcus, pneumococcus, influenza, or Covid-19) were performed at the same time as the study injections*”) and we added a sentence in the discussion section of the revised (R2) version of the manuscript.

**Reviewer #2**

**The terms DTaP 2 and DTaP 3 referred to the number of acellular pertussis components in the hexavalent vaccine. A large number of vaccine studies have used correlates of immunity aaginst pertussis - also accepted by vaccine regulatory bodies. Considering that it is re-emerging in a big way, I think this should have been looked at**.

This remark is very relevant: the issue of pertussis re-emergence is indeed concerning. Alas, when we designed this study, we decided not to analyze anti-pertussis antibody response. We may regret this, but we cannot produce analyses from data we purposely did not collect.

On this matter, we have let in the revised (R2) version of the manuscript the sentence we had already introduced in the R1 revised version to acknowledge this (“*In addition, we did not assess the immune response to pertussis, which is currently reemerging; studies reported a lower immune response (68% seroprotection) for this pathogen after post-transplant vaccination [ref. 23], although the lack of clear correlates of protection for pertussis had led us not to assess the immunogenicity of this valence*”).
